# Semi-Synthesis of Small Molecules of Aminocarbazoles: Tumor Growth Inhibition and Potential Impact on p53

**DOI:** 10.3390/molecules26061637

**Published:** 2021-03-15

**Authors:** Solida Long, Joana B. Loureiro, Carla Carvalho, Luís Gales, Lucília Saraiva, Madalena M. M. Pinto, Ploenthip Puthongking, Emília Sousa

**Affiliations:** 1 Laboratory of Organic and Pharmaceutical Chemistry (LQOF), Department of Chemical Sciences, Faculty of Pharmacy, University of Porto, Rua de Jorge Viterbo Ferreira, 228, 4050-313 Porto, Portugal; solidachhann@gmail.com or madalena@ff.up.pt (M.M.M.P.); 2 Laboratory of Microbiology (LAQV/REQUIMTE), Department of Biological Sciences, Faculty of Pharmacy, University of Porto, Rua de Jorge Viterbo Ferreira, 228, 4050-313 Porto, Portugal; up201407524@ff.up.pt (J.B.L.); pg32852@alunos.uminho.pt (C.C.); 3 Institute for the Biomedical Science Abel Salazar (ICBAS), University of Porto, Rua de Jorge Viterbo Ferreira, 228, 4050-313 Porto, Portugal; lgales@ibmc.up.pt; 4 Instituto de Biologia Molecular e Celular (i3S-IBMC), University of Porto, Rua de Jorge Viterbo Ferreira, 228, 4050-313 Porto, Portugal; 5 Interdisciplinary Centre of Marine and Environmental Research (CIIMAR), 4450-208 Matosinhos, Portugal; 6 Department of Pharmaceutical Chemistry, Faculty of Pharmaceutical Sciences, Khon Kean University, Khon Kean 40002, Thailand; pploenthip@kku.ac.th

**Keywords:** aminocarbazoles, heptaphylline, alkaloids, tumor, p53, mutant

## Abstract

The tumor suppressor p53 is inactivated by mutation in approximately 50% of human cancers. Small molecules that bind and stabilize those mutants may represent effective anticancer drugs. Herein, we report the tumor cell growth inhibitory activity of carbazole alkaloids and amino derivatives, as well as their potential activation of p53. Twelve aminocarbazole alkaloids were semi-synthesized from heptaphylline (**1**), 7-methoxy heptaphylline (**2**), and 7-methoxymukonal (**3**), isolated from *Clausena harmandiana*, using a reductive amination protocol. Naturally-occurring carbazoles 1–3 and their amino derivatives were evaluated for their potential effect on wild-type and mutant p53 activity using a yeast screening assay and on human tumor cell lines. Naturally-occurring carbazoles 1–3 showed the most potent growth inhibitory effects on wild-type p53-expressing cells, being heptaphylline (**1**) the most promising in all the investigated cell lines. However, compound **1** also showed growth inhibition against non-tumor cells. Conversely, semi-synthetic aminocarbazole 1d showed an interesting growth inhibitory activity in tumor cells expressing both wild-type and mutant p53, exhibiting low growth inhibition on non-tumor cells. The yeast assay showed a potential reactivation of mutant p53 by heptaphylline derivatives, including compound **1d**. The results obtained indicate that carbazole alkaloids may represent a promising starting point to search for new mutp53-reactivating agents with promising applications in cancer therapy.

## 1. Introduction

**Figure 1:**
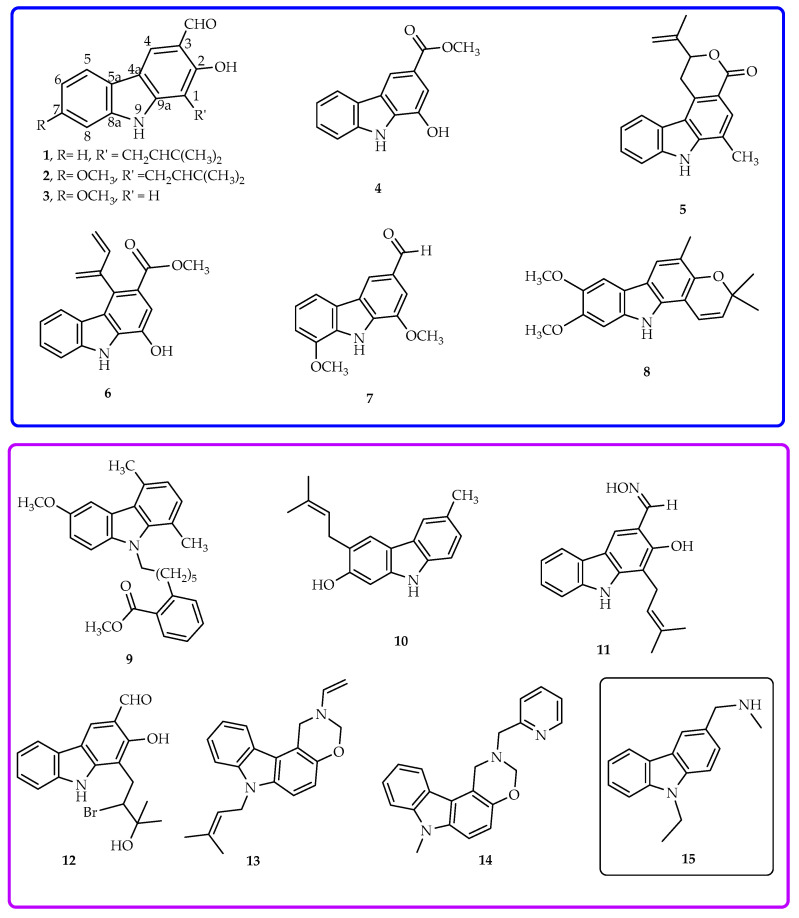
Some examples of carbazole alkaloids: natural isolated carbazoles **1–8**, semi-synthetic analogues **9**–**14**, and derivative **15**.

Carbazole alkaloids natural products are mostly isolated from higher plants of Rutaceae family and major components of the *Clausena* genus [1,2]. With the isolation of carbazole core from coal tar in 1872 [3] and the description of the antimicrobial murrayanine in 1965 [4], the interest on these alkaloids began. Since then, natural-occurring carbazole alkaloids have been reported to exhibit a broad pharmacological profile, including activities such as antitumor (i.e., heptaphylline **1**, Figure 1) [5], 7-methoxy-heptaphylline (**2**) [6], 2-hydroxy-7-methoxy-9*H*-carbazole-3-carbaldehyde or 7-methoxy-mukonal (**3**) [7]), antiplasmodial (i.e., compounds **1** [8] and **3** [7]), antiplatelet aggregation, and vasorelaxing (i.e., clausine E (**4**) [9]), antibacterial (i.e., clausamine B (**5**), clausine F (**6**) [10], and clausenal (**7**) [11]), antifungal (i.e., compound **7** [11]), and antidiabetic (i.e., koenidine (**8**) [12]). Recently, heptaphylline (**1**) was reported to induce apoptosis in a human colon adenocarcinoma cell line [13] and was considered a promising model for new anticancer drugs. In addition, the carbazole nucleus can be easily functionalized mainly at positions 3, 6, and 9 to obtain bioactive derivatives [2,6]. For instance, analogue **9** was reported with anti-Alzheimer properties [14], and compound **10** with activity against human immunodeficiency virus, type-1 (HIV-1) [15]. Derivatives **11** and **12** of carbazole **1** were found to exhibit strong cytotoxicity against NCl-H187 and KB cells, 138 fold stronger than ellipticine standard [6,16,17,18,19] while *N*-substituted derivatives, such as compounds **13** and **14,** were reported as tumor growth inhibitors against leukemia cells ECM, Jurkat, and Raji with concentration that induces 50% of growth inhibition (IC_50_) values around 12 µM [17].

Inhibitors of tumor cell lines have been associated to several mechanisms, one of which being through the p53 pathway. The tumor suppressor protein p53 is a transcription factor that plays a key role in the prevention of cancer development, mainly due to its major role in cellular events such as apoptosis, cell cycle progression, and DNA repair [20,21]. However, over 50% of p53 proteins present missense mutations, generating a defective protein in high levels in cells due to the impairment of MDM2 (murine doble minute 2) mediated negative feedback, which is responsible for p53 degradation. p53 Protein is known as the guardian of the genome because one of the most important p53 functions is the ability to activate apoptosis and the disruption of this process can be correlated with tumor progression and chemoresistance [22]. In tumor cells, the restoration of p53 function has shown to be highly effective against tumor cells, thus reactivating mutant p53 has been a goal in anticancer drug development [23]. Some small molecules in the group of carbazole alkaloids have been reported to reactivate mutant p53 by restoration the wild-type (wt) structure/function [24,25]. For example, PhiKan083 (**15**), an amino derivative of the carbazole, emerged from an in silico screening [26] and was reported as a small molecule for restoration of wild-type like p53 conformation by targeting Y220 mutation [26,27]. This derivative **15** established electrostatic and hydrogen bonding interactions with residues of Y220 which gave additional stability to Y220 mutant p53. This particular mutation creates a druggable surface crevice and PhiKan083 (**15**) binds to this crevice and stabilizes the structure of this mutant p53 [27,28]. Up to date, none of the natural isolated carbazole alkaloids or their chemical modified ana was reported to have effect on p53 mutants. Herein, a series of semi-synthetic aminocarbazoles was synthesized from naturally-occurring heptaphylline (**1**), and their tumor cell growth inhibition and potential activity on p53 were studied.

## 2. Results and Discussion

### 2.1. Semi-Synthesis of Aminocarbazole Alkaloids by Direct Reductive Amination

The reaction of carbonyl groups, aldehydes, or ketones with amines in the present of reducing agents to give corresponding amines, known as reductive amination (of carbonyl compounds) or reductive alkylation (of amine compounds) is one of the most useful and important methods in the synthesis of different kind of amines as well as a powerful reaction to obtain drug candidates [29]. The choice and understanding of the reducing agent are essential for the selection of the reaction conditions. Sodium triacetoxyborohydride [NaBH(OAc)_3_, STAB] was reported as the most powerful reducing agent in direct reductive amination due to its stability and safety.

Reductive aminations of **1**, **2**, and **3** were performed in an one-pot conversion of their carbonyl group in the present of STAB with two different solvents—dried tetrahydrofuran (THF) or dried 1,2-dichloroethane (DCE) with selected amines precursors present in inhibitors of p53:MDM2 interaction. The reaction mixtures were stirred under nitrogen gas until no further developments to yield aminocarbazole alkaloids derivatives **1a**–**1e**, **2a**–**2f**, and **3a** (Table 1). Products were treated with different work-up procedures before purification, as described in the experimental section.

**Figure 2:**
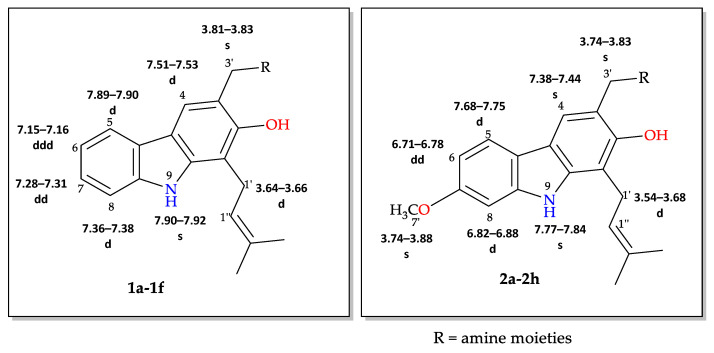
Key protons for compounds **1a**–**1f** and **2a**–**2h**.

Generally, in the present of STAB, the reactions of **1**, **2** and **3** with primary amines yield secondary amines (entry 3–5 and 8–10), via imine intermediates, and the reaction with secondary amines yield tertiary amines (entry 1–2, 6–7 and 11–12), via enamine intermediates. The final products were categorized into 3 groups, alkylated linear aminocarbazoles, compounds **1a**, **2a**, and **3a**, heterocyclic aminocarbazoles, compounds **1b**, **2b**, and **2f**, and halogenated aminocarbazoles, compounds **1c**–**1e** and **2c**–**2e**. The reactions mostly showed no further development between 3–10 days. All the reactions required long reaction times due to the steric hindered of the hydroxyl at position 2 and/or prenyl group at position 1. Aminocarbazoles modified from **2**, compounds **2a**–**2f**, and from **3**, compound **3a**, required longer reaction time than those derived from **1**, compounds **1a**–**1e**, in both solvent conditions. These longer times should be related to the effect of the methoxy electron donating group at position 7. The reductive amination with primary amines was faster than with secondary amines (entry 4–6 and 8–10). Reactions performed in DCE required shorter times and produced higher yields (3–5 days, 34–90%) compared to those performed in THF (3–10 days, 13–51%), and these results are in agreement with previous reports [30]. All the compounds were confirmed by one- and two-dimensional NMR and high-resolution mass spectrometry. The chemical shift of protons and carbons of **1**, **2**, and **3** were accordance to the literature [31,32]. The analysis of (+) HRMS-ESI, ^1^H, ^13^C NMR, HSQC, HMBC, and X-ray crystallographic data (in case of compound **1b**) revealed the success of the reductive amination to produce amine derivatives. Compounds derived from **1** and **2**, showed the proton H-1′ signal as a doublet (d) with chemical shift δ values of *c.a* 3.50–3.68 ppm while proton H-3′ signal appeared as a singlet with δ values *c.a* 3.74–3.83 ppm. The proton signal of one of the methyl groups of the prenyl substituent appeared as a singlet at *c.a* 1.90 ppm while another methyl signal was presented as a narrow doublet at *c.a* 1.76 ppm due to the correlation to the H-1”. Aminocarbazoles derived from **1**, compounds **1c**, **1d**, **1e**, and compounds derived from **2**, compounds **2c**, **2d**, and **2e**, presenting secondary amine moieties showed the chemical shift of proton H-5′ with δ values *c.a* 4.14 and 4.05 ppm, respectively appearing as singlets (see in experimental section). We also summary the key protons of amine derivatives obtained from substrate **1** and **2** as shown in Figure 2. For semi-synthetic derivatives from **1**, the signals of protons H-5, appeared as doublets with δ values *c.a* 7.89–7.90 ppm, H-6 as doublet-doublet-doublet with δ values *c.a* 7.15–7.16 ppm, H-7 as doublet-doublet δ values *c.a* 7.28–7.31 ppm, and H-8 as doublet with δ values *c.a* 7.36–7.38 ppm, respectively. For semi-synthetic derivatives of **2** and **3**, having a methoxy group at position 7, the proton signals of H-6 appeared as doublet-doublet with δ values *c.a* 6.71–6.78 ppm, and H-8 as doublets with δ values *c.a* 6.82–6.88 ppm, respectively.

**Figure 3:**
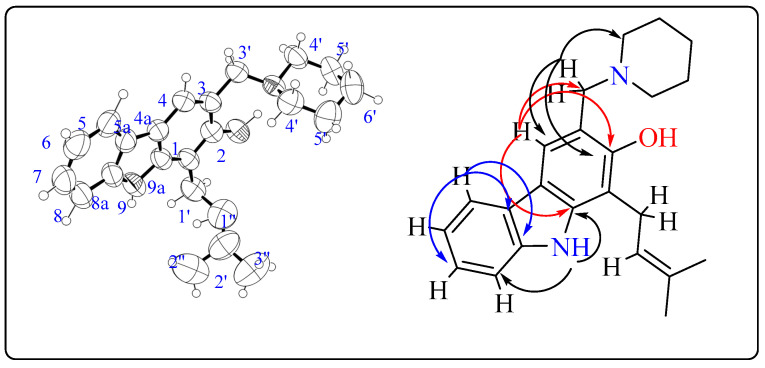
Ortep view of compound **1b** and its key HMBC correlations.

Compound **1b** was obtained as in a crystal form in the mixture of methanol and ethyl acetate. The X-ray crystallographic representation of compounds **1b** is presented in Figure 3. The Ortep diagram confirmed the structure of **1b**, and the analyses of HSQC and HMBC provided the correlations of compound **1b** depicted (Figure 3). The key proton H-4 showed correlations with C-3′, C-2, and C-9a while H-5 showed correlations with C-7 and C-8a. The proton of the indole group H-9 showed correlations with C-8 and C-9a while proton H-3′ showed correlations with C-4′, C-2, and C-4.

### 2.2. Aminocarbaozoles Exhibit Tumor Growth-Inhibitory Effect

**Figure 4:**
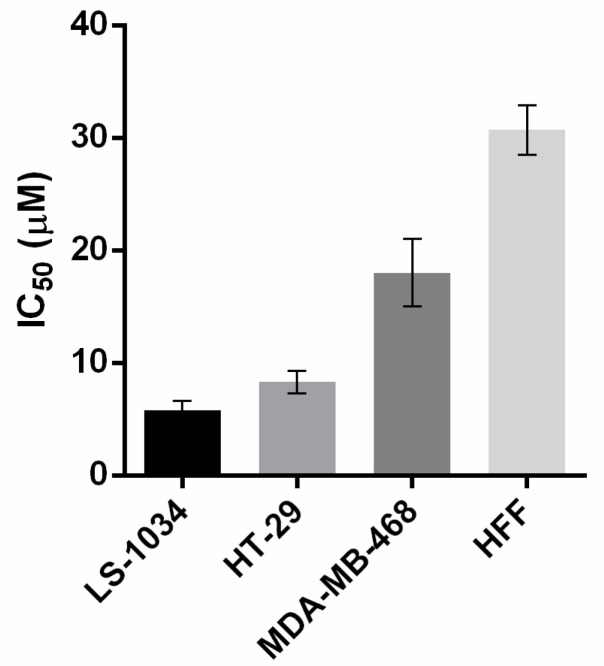
IC_50_ values of compound **1d** in tumor cells expressing mutant p53 and in non-tumorigenic cells (HFF). The concentration that induces 50% of growth inhibition (IC_50_) was determined by SRB assay after 48 h treatment. Data are mean ± SEM of 3–4 independent experiments.

The tumor cell growth inhibitory potential of aminocarbazoles was ascertained in human colon adenocarcinoma HCT116 and melanoma A375 cell lines expressing wild-type (wt) p53, in colorectal HT-29-R273H, HuH-7-Y220, SW837-R248W, MDA-MB-468-R273H, SF-268-R273H, and LS1034-G245S cell lines expressing mutant p53. All naturally-occurring carbazoles showed a strong inhibitory effect, while their aminocarbazoles **1a**–**1e**, **2b**–**2e**, and **3a** showed moderate inhibitory effects (IC_50_ range between 4.5 to > 50 µM) in all tested cell lines (Table 2) in which heptaphylline (**1**) demonstrated marked antiproliferative activity. A promising activity could be observed with compound **1d**, which displayed and evident growth inhibitory activity in cell lines expressing mutant p53, particularly in HT-29, MDA-MB-468, and LS-1034, and a significantly lower activity in non-tumorigenic cells (Figure 4).

### 2.3. Evaluation of Aminocarbazoles Potential Activation of p53 Using a Yeast-Based Screening Assay

Compounds containing a carbazole scaffold have been identified and tested against a certain mutation of p53 and showed to stabilize the mutant in which the carbazole ring system is sandwiched in hydrophobic side chains [26]. In this work, to identify small molecules of aminocarbazoles that could restore p53 pathway signalling, three aminocabazoles derived from heptaphylline (**1b–d**) and the natural compound **1** were tested for their ability to activate wt or mutant p53, using a previously developed yeast-based screening assay [33]. Among the tested compounds in cells lines, four were selected due to their highest growth inhibitory activity in tumor cells. In the yeast assay, yeast cells expressing wt p53 present a marked growth inhibition, which is reduced or abolished in case of mutant p53. Compounds able to activate wt p53 or to restore the wild-type-like activity to mutant p53 will increase the growth inhibition induced by expression of the human protein in yeast [33]. Yeast cells expressing mutant or wt p53, and control yeast (transformed with the empty vector) were treated with 10 μM of each compound and its impact on yeast growth inhibition was evaluated. All the compounds tested were able to reactivate at least two of the mutant p53 forms studied, increasing their yeast growth inhibitory effect (Table 3). Among the compounds tested, only compound **1b** was able to also activate wt p53 in yeast. It is of note that compounds **2** and **3** were cytotoxic in control yeast, and therefore these natural products were excluded from the assay.

Percentage of p53 reactivation induced by heptaphylline derivatives. Data were normalized to the percentage of wtp53 growth inhibitory effect in yeast cells. Yeast expressing human mutant p53 or wt p53 were treated for 42 h with the indicated compound. Results correspond to the percentage of wt p53-induced growth inhibition re-established by compounds in yeast expressing mutant p53. Data are mean ± SEM of 3–6 independent experiments. Dashes represent a reactivation effect lower than 30%.

Interestingly, in the yeast-screening assay, compound **1d** demonstrated the most promising activity in mutations involving codon 245 of p53, namely G245D and G245S. In fact, through the antiproliferation assay, compound **1d** displayed its greatest antiproliferative activity in LS-1034 expressing mutant p53 G245S.

## 3. Materials and Methods

### 3.1. Isolation

The root bark of *Clausena harmandiana* (Pierre) Guillaumin (Rutaceae) was collected in Khon Kaen province, Thailand, in June 2016. Authentication was identified by comparison with the herbarium specimen at the Faculty of Science, Khon Kaen University. The identified voucher specimen (KKU No. 21145) was deposited at Faculty of Pharmaceutical Sciences, Khon Kaen Univerisity, Thailand. The root barks (2.29 kg) were air-dried, ground, and sequentially extracted at room temperature for overnight with dichloromethane (4 times). The extracts were evaporated in vacuo to obtain crude dichloromethane extract (140 g). The crude dichloromethane was isolated by open column chromatography on silica gel 60 and subsequently eluted with a gradient of *n-*hexane and ethyl acetate (EtOAc) to give **1** (310 mg; 1.4 × 10^−2^ of dry weight), **2** (340 mg; 1.5 × 10^−2^ of dry weight), and **3** (170 mg; 0.7 × 10^−2^ of dry weight). All isolated compounds were structurally elucidated by comparison with the authentic samples, which were identical in all respects [34].

### 3.2. Purity Determination by HPLC-DAD

The HPLC system consisted of Shimadzu LC-20AD pump, equipped with a Shimadzu DGV-20A5 degasser, a Rheodyne 7725i injector fitted with a 20 µL loop, and a SPD-M20A DAD detector (Kyoto, Japan). Data acquisition was performed using Shimadzu LCMS Lab Solutions software, version 3.50 SP2. The column used in this study was ACE-C18 (150 × 4.6 mm I.D., particle size 5 µm) manufactured by Advanced Chromatography Technologies Ltd. (Aberdeen, Scotland, UK). The mobile phase composition was water and methanol (2:8 *v*/*v*; 0.1% triethylamine), all were HPLC grade solvents obtained from Merck Life Science S.L.U. (Darmstadt, Germany). The flow rate was 1.0 mL/min and the UV detection wavelength was 312 nm. Analyses were performed at 27 °C in an isocratic mode. Peak purity index was determined by total peak UV-Vis spectra between 210–800 nm with a step of 4 nm. The percentage is indicated at each compound and detailed data is given in Supplementary Material.

### 3.3. General Semi-Synthesis of the Aminocarbazole Derivatives of Heptaphylline *(**1**)*, 7-Methoxyheptaphylline *(**2**)*, and 7-Methoxymukonal *(**3**)*

Naturally carbazole alkaloid heptaphylline (**1**, 40 mg, 0.132 mmol) or 7-methoxyheptaphylline (**2**, 41 mg, 0.132 mmol) or 7-methoxymukonal (**3**, 32 mg, 0.132 mmol) and the amine precursors such as *N,N,N*-trimethyl-1,3-propanediamine (0.52 mL, 3.5 mmol, 27 equiv.) for compounds **1a**, **2a**, and **3a**, or piperidine (0.1 mL, 3.5 mmol, 27 equiv.) for compounds **1b** and **2b**, or 4-chlorobenzylamine (0.081 mL, 0.66 mmol, 5 equiv.) for compounds **1c** and **2c**, or 4-fluorobenzylamine (0.075 mL, 0.66 mmol, 5 equiv.) for compounds **1d** and **2d**, or 4-bromobenzylamine (0.083 mL, 0.66 mmol, 5 equiv.) for compounds **1e** and **2e**, or 1,2,3,4-tetrahydroisoquinoline (28 mg, 0.184 mmol, 1.4 equiv.) for compound **2f**, were dissolved in dried THF or dried DCE, and added to the reaction mixture of the STAB (84.8 mg, 0.36 mmol, 3 equiv.). After adding the acetic acid (8.2 µL, 0.132 mmol, 1equiv.), the mixture was stirred at r.t under N_2_ no longer than 14 days. For monitoring the synthesis of aminocarbazole derivatives by TLC, two chromatographic systems were used: *n*-hexane:EtOAc 7:3 and CHCl_3_:(CH_3_)_2_CO: TEA 100:0.1 for amine. The crude product obtained from the reactions was subjected to different work-up strategies. After reaction of compounds **1a, 1b, 2a**, **2b**, **2f**, and **3a** the crudes were extracted with CHCl_3_ (3 × 50 mL), then solid phase extraction (SPE) through cation exchange cartridge Discovery^®^ DSC-SCX (Supelco, Bellefonte, Philadelphia, PA, USA) using 1% NH_3_ in CH_3_OH. The basic fractions were purified on flash column using Hexane:EtOAc; 7:3. For compounds **1c**, **1d**, **1e** and **2c**, **2d**, **2e**, after reaction, the crudes extracts were treated with 5% of NaOH in CHCl_3_ (3 × 50 mL) to remove excess STAB, the organic phases were treated with 5M HCl in CHCl_3_ to remove excess amines. Then, the aqueous phases were treated with 20% of NaOH in CHCl_3_. The combination of organic phases was subjected to SPE through cation exchange cartridge Discovery^®^ DSC-SCX using 1% NH_3_ in CH_3_OH. The basic fractions were purified on flash column using *n*-hexane:EtOAc 7:3.

*3-{[(3-(Dimethylamino)propyl)(methyl)amino]methyl}-1-(3-methylbut-2-en-1-yl)-9H-carbazol-2-ol* (**1a**). 25.2 mg; 49%; greenish yellow solid; purity HPLC-DAD 93.8%; mp: 160.3–161.2 °C; IR (KBr) *v_max_* cm^−1^: 3319, 2924, 1632, 1439, 1374, 1205, 740; ^1^H NMR (CDCl_3_, 300 MHz) δ: 7.92 (1H, br, NH), 7.89 (1H, d, *J* = 7.8 Hz, H-5), 7.53 (1H, s, H-4), 7.37 (1H, d, *J* = 7.9 Hz, H-8), 7.28 (1H, ddd, *J* = 8.5, 7.0, 1.7 Hz, H-7), 7.15 (1H, dt, *J* = 7.5, 1.1 Hz, H-6), 5.36 (1H, ddd, *J* = 6.8, 5.4 and 1.4 Hz, H-1”), 3.84 (2H, s, H-3′), 3.64 (2H, d, *J* = 6.7 Hz, H-1′), 2.56 (2H, t, *J* = 7.5 Hz, H-4′), 2.33 (3H, s, H-4″), 2.36 (2H, t, *J* = 7.5 Hz, H-6′), 2.23 (6H, s, H-6″), 1.90 (3H, s, H-2″), 1.76 (3H, d, *J* = 1.2 Hz, H-3”); ^13^C NMR (CDCl_3_, 75 MHz): 154.1 (C-2), 140.0 (C-8a), 139.4 (C-9a), 132.9 (C-2′), 124.0 (C-7), 123.8 (C-5a), 122.7 (C-1″), 119.3 (C-6), 119.1 (C-5), 117.6 (C-4), 115.4 (C-4a), 115.3 (C-3), 110.4 (C-8), 109.3 (C-1), 62.3 (C-3′), 57.4 (C-6′), 54.8 (C-4′), 45.3 (C-6″), 41.1 (C-4″), 25.8 (C-3″), 25.0 (C-5′), 18.1 (C-2″); HRMS-ESI *m/z* 380.2697 (M + H)^+^ (calculate for C_24_H_33_N_2_O, 379.2624).

*1-(3-Methylbut-2-en-1-yl)-3-(piperidin-1-ylmethyl)-9H-carbazol-2-ol* (**1b**). 39.2 mg; 90%; greenish yellow oil; purity HPLC-DAD 95.8%; mp: 155.0–155.7 °C; IR (KBr) *v_max_* cm^−1^: 3425, 2923, 1633, 1438, 1374, 1222, 741; ^1^H NMR (CDCl_3_, 300 MHz) δ: 7.90 (1H, br, NH), 7.89 (1H, d, *J* = 7.7 Hz, H-5), 7.51 (1H, s, H-4), 7.36 (1H, d, *J* = 7.9 Hz, H-8), 7.29 (1H, ddd, *J* = 8.0, 6.8, 1.1 Hz, H-7), 7.15 (1H, dd, *J* = 7.1, 1.1 Hz, H-6), 5.37 (1H, ddd, *J* = 6.8, 5.1 and 1.3 Hz, H-1”), 3.81 (2H, s, H-3′), 3.65 (2H, d, *J* = 6.8 Hz, H-1′), 2.55 (4H, m, H-4′), 1.90 (3H, s, H-2″), 1.76 (3H, d, *J* = 1.2 Hz, H-3”), 1.65 (4H, m, H-5′), 1.51 (2H, m, H-6′); ^13^C NMR (CDCl_3_, 75 MHz): 154.3 (C-2), 140.0 (C-8a), 139.3 (C-9a), 132.9 (C-2′), 124.0 (C-7), 123.9 (C-5a), 122.7 (C-1”), 119.2 (C-6), 119.0 (C-5), 117.6 (C-4), 117.6 (C-3), 115.3 (C-4a), 115.0 (C-1), 110.3 (C-8), 62.8 (C-3′), 53.7 (C-4′), 25.8 (C-5′), 25.7 (C-2″), 24.1 (C-6′), 23.8 (C-1′), 18.1 (C-3″); HRMS-ESI *m/z* 349.2270 (M + H)^+^ (calculated for C_23_H_28_N_2_O, 349.2280).

*3-{[(4-Chlorobenzyl)amino]methyl}-1-(3-methylbut-2-en-1-yl)-9H-carbazol-2-ol* (**1c**). 21.1 mg; 49%; greenish yellow solid; purity HPLC-DAD 99.7%; mp: 98.3–99.6 °C; IR (KBr) *v_max_* cm^−1^: 3420, 2917, 1635, 1463, 1378, 729, 668; ^1^H NMR (CDCl_3_, 300 MHz) δ: 7.93 (1H, br, NH), 7.89 (1H, d, *J* = 7.8 Hz, H-5), 7.55 (1H, s, H-4), 7.38 (1H, d, *J* = 7.8 Hz, H-8), 7.34–7.29 (2H, m, H-6”), 7.28 (1H, ddd, *J* = 7.6, 5.1, and 3.4 Hz, H-7), 7.16 (1H, ddd, *J* = 9.1, 6.8 and 1.2 Hz, H-6), 7.10–6.99 (2H, m, H-7”), 5.36 (1H, ddd, *J* = 6.8, 4.1 and 1.4 Hz, H-1”), 4.14 (2H, s, H-3′), 3.84 (2H, s, H-5′), 3.66 (2H, d, *J* = 6.7 Hz, H-1′), 1.91 (3H, s H-2″), 1.76 (3H, d, *J* = 1.2 Hz, H-3”); ^13^C NMR (CDCl_3_, 75 MHz): 154.1 (C-2), 140.1 (C-9a), 139.4 (C-8a), 134.1 (C-6′), 133.0 (C-2′), 130.1 (C-6”), 130.0 (C-8′), 124.1 (C-7), 124.0 (C-5a), 123.9 (C-2″), 122.5 (C-1”), 119.3 (C-6), 119.1 (C-5), 117.7 (C-4), 115.6 (C-4a), 115.4 (C-7”), 115.2 (C-3), 110.4 (C-8), 109.8 (C-1), 52.5 (C-3′), 51.8 (C-5′), 23.9 (C-1′), 25.8 (C-2″), 18.1 (C-3”); HRMS-ESI *m/z* 405.1782 (M + H)^+^ (calculated for C_25_H_25_N_2_ClO, 405.1733).

*3-{[(4-Fluorobenzyl)amino]methyl}-1-(3-methylbut-2-en-1-yl)-9H-carbazol-2-ol* (**1d**). 38.5 mg; 42%; greenish yellow solid; purity HPLC-DAD 98.8%; mp: 96.1–96.5 °C; IR (KBr) *v_max_* cm^−1^: 3421, 2923, 1633, 1438, 1375, 1222, 741; ^1^H NMR (CDCl_3_, 300 MHz) δ: 7.92 (1H, br, NH), 7.89 (1H, d, *J* = 7.5 Hz, H-5), 7.55 (1H, s, H-4), 7.36 (1H, d, *J* = 7.5 Hz, H-8), 7.31 (2H, m, H-6”), 7.30 (1H, ddd, *J* = 8.5, 7.0, 1.7 Hz, H-7), 7.18 (2H, dt, *J* = 8.5 2.5 Hz, H-7″), 7.14 (1H, ddd, *J* = 8.5, 7.0, 1.1 Hz, H-6), 5.36 (1H, ddd, *J* = 6.9, 4.6, and 1.5 H-1”), 4.14 (2H, s, H-3′), 3.84 (2H, s, H-5′), 3.66 (2H, d, *J* = 6.7 Hz, H-1′), 1.91 (3H, d, *J* = 0.6 Hz, H-2″), 1.77 (3H, d, *J* = 1.2 Hz, H-3”); ^13^C NMR (CDCl_3_, 75 MHz) 160.5 (C-7′), 154.3 (C-2), 140.2 (C-9a), 139.4 (C-8a), 136.9 (C-6′), 133.1 (C-2′), 130.1 (C-6”), 124.1 (C-7), 122.6 (C-1”), 119.7 (C-6), 119.6 (C-5), 117.8 (C-4), 115.9 (C-3), 115.8 (C-4a), 115.7 (C-5a), 115.5 (C-7”), 109.6 (C-1), 110.3 (C-8), 52.4 (C-3′), 51.8 (C-5′), 23.7 (C-1′), 25.8 (C-2″), 18.1 (C-3”); HRMS-ESI *m/z* 389.2023 (M + H)^+^ (calculated for C_25_H_25_N_2_FO, 389.2062).

*3-{[(4-Bromobenzyl)amino]methyl}-1-(3-methylbut-2-en-1-yl)-9H-carbazol-2-ol* (**1e**). 40.66 mg; 64%; greenish yellow solid; purity HPLC-DAD 98.0%; mp: 145.6–146.4 °C; IR (KBr) *v_max_* cm^−1^: 3319, 2924, 1632, 1439, 1374, 1205, 740; ^1^H NMR (CDCl_3_, 300 MHz) δ: 7.92 (1H, br, NH), 7.90 (1H, d, *J* = 7.9 Hz, H-5), 7.55 (1H, s, H-4), 7.49 (2H, m, H-7”), 7.38 (1H, d, *J* = 8.0 Hz, H-8), 7.31 (1H, ddd, *J* = 8.3, 7.5, 1.2, H-7), 7.21 (2H, dt, *J* = 8.5 2.5 Hz, H-6″), 7.16 (1H, ddd, *J* = 8.5, 7.0, 1.1 Hz, H-6), 5.36 (1H, m, H-1”), 4.14 (2H, s, H-3′), 3.83 (2H, s, H-5′), 3.66 (2H, d, *J* = 6.8 Hz, H-1′), 1.91 (3H, s, H-2″), 1.77 (3H, d, *J* = 1.1 Hz, H-3”); ^13^C NMR (CDCl_3_, 75 MHz) 153.7 (C-2), 140.2 (C-9a), 139.4 (C-8a), 138.9 (C-6′), 133.1 (C-2′), 131.8 (C-7”), 130.4 (C-6”), 124.5 (C-7), 123.6 (C-5a), 122.5 (C-1”), 121.5 (C-7′), 119.5 (C-6), 119.3 (C-5), 117.9 (C-4), 115.7 (C-3), 115.3 (C-4a), 109.8 (C-1), 110.6 (C-8), 52.5 (C-3′), 51.7 (C-5′), 23.6 (C-1′), 25.8 (C-2″), 18.1 (C-3″); HRMS-ESI *m/z* 447.471051 (M + H)^+^ (calculated for C_25_H_25_N_2_BrO, 446.0994).

*3-{[(3-(Dimethylamino)propyl)(methyl)amino]methyl}-7-methoxy-1-(3-methylbut-2-en-1-yl)-9H-carbazol-2-ol* (**2a**). 27.8 mg; 51.2%; greenish yellow solid; purity HPLC-DAD 96.8%; mp: 162.7–163.8 °C; IR (KBr) *v_max_* cm^−1^: 3319, 2924, 1632, 1439, 1374, 1205, 740; ^1^H NMR (CDCl_3_, 300 MHz) δ: 7.84 (1H, br, NH), 7.74 (1H, d, *J* = 8.5 Hz, H-5), 7.42 (1H, s, H-4), 6.88 (1H, d, *J* = 2.1 Hz, H-8), 6.78 (1H, dd, *J* = 8.5, 2.3 Hz, H-6), 5.35 (1H, m, H-1”), 3.88 (3H, s, H-7′), 3.82 (2H, s, H-3′), 3.62 (2H, d, *J* = 6.7 Hz, H-1′), 2.56 (2H, t, *J* = 7.3 Hz, H-4′), 2.42 (2H, t, *J* = 7.5 Hz, H-6′), 2.32 (9H, s, H-4″ and 6”), 1.89 (3H, s, H-2″), 1.82 (2H, t, *J* = 7.5 Hz, H-5′), 1.76 (3H, d, *J* = 1.1 Hz, H-3”); ^13^C NMR (CDCl_3_, 75 MHz) 157.9 (C-7), 153.1 (C-2), 140.6 (C-8a), 139.9 (C-9a), 132.9 (C-2′), 122.7 (C-1″), 119.7 (C-5), 117.9 (C-5a), 116.9 (C-4), 115.6 (C-4a), 115.1 (C-3), 109.4 (C-1), 107.7 (C-6), 95.0 (C-8), 62.3 (C-3′), 57.1 (C-6′), 55.7 (C-4′), 44.8 (C-6″), 41.1 (C-4″), 25.9 (C-3″), 24.2 (C-5′), 18.1 (C-2″); HRMS-ESI *m/z* (calculated for C_25_H_35_N_2_O_2_, 409.2729).

*7-Methoxy-1-(3-methylbut-2-en-1-yl)-3-(piperidin-1-ylmethyl)-9H-carbazol-2-ol* (**2b**). 19.56 mg; 39%; greenish yellow oil; purity HPLC-DAD 98.3%; mp: 157.8–159.4 °C; IR (KBr) *v_max_* cm^−1^: 3397, 2919, 1652, 1449, 1361, 1035, 817, 784; ^1^H NMR (CDCl_3_, 300 MHz) δ: 7.84 (1H, br, NH), 7.74 (1H, d, *J* = 8.5 Hz, H-5), 7.41 (1H, s, H-4), 7.88 (1H, d, *J* = 2.2 Hz, H-8), 6.77 (1H, dd, *J* = 8.5, 2.2 Hz, H-6), 5.56 (1H, m, H-1”), 3.88 (3H, s, H-7′), 3.79 (2H, s, H-3′), 3.63 (2H, d, *J* = 6.7 Hz, H-1′), 2.63 (4H, m, H-4′), 1.90 (3H, s, H-2″), 1.76 (3H, d, *J* = 1.2 Hz, H-3”), 1.65 (4H, m, H-5′), 1.48 (2H, m, H-6′); ^13^C NMR (CDCl_3_, 75 MHz): 157.8 (C-7), 153.4 (C-2), 140.5 (C-8a), 139.9 (C-9a), 132.9 (C-2′), 122.8 (C-1″), 119.7 (C-5), 117.9 (C-5), 116.9 (C-4), 115.5 (C-4a), 114.8 (C-1), 107.6 (C-6), 94.9 (C-8), 62.8 (C-3′), 53.7 (C-4′), 25.8 (C-5′), 25.7 (C-2″), 24.1 (C-6′), 23.8 (C-1′), 18.1 (C-3″); HRMS-ESI *m/z* 379.2382 (M + H)^+^ (calculated for C_24_H_30_N_2_O_2_, 378.2307).

*3-{[(4-Chlorobenzyl)amino]methyl}-7-methoxy-1-(3-methylbut-2-en-1-yl)-9H-carbazol-2-ol* (**2c**). 19.60 mg; 34%; greenish yellow oil; purity HPLC-DAD 97.9%; mp: 109.8–110.2 °C; IR (KBr) *v_max_* cm^−1^: 3318, 2917, 1617, 1492, 1361, 1016, 801, 669; ^1^H NMR (CDCl_3_, 300 MHz) δ: 7.78 (1H, br, NH), 7.75 (1H, d, *J* = 8.5 Hz, H-5), 7.44 (1H, s, H-4), 7.32 (dt, 2H, *J* = 9.4, 3.0 Hz, H-6”), 7.26 (m, 2H, H-7”), 6.89 (1H, d, *J* = 2.1 Hz, H-8), 6.78 (1H, dd, *J* = 8.5, 2.2 Hz, H-6), 5.35 (1H, m, H-1”), 4.12 (2H, s, H-3′), 3.88 (3H, s, H-7′), 3.82 (2H, s, H-5′), 3.64 (2H, d, *J* = 6.7 Hz, H-1′), 1.90 (3H, s, H-2″), 1.76 (3H, d, *J* = 1.1 Hz, H-3”); ^13^C NMR (CDCl_3_, 75 MHz): 157.9 (C-7), 153.2 (C-2), 140.5 (C-8a), 140.0 (C-9a), 137.1 (C-6′), 133.3 (C-8′), 133.0 (C-2′), 129.8 (C-6”), 128.8 (C-7″), 122.6 (C-1″), 119.8 (C-5), 117.9 (C-5a), 116.9 (C-4), 115.7 (C-3), 115.5 (C-4a), 109.8 (C-1), 107.6 (C-6), 95.0 (C-8), 52.7 (C-3′), 51.8 (C-5′), 23.9 (C-1′), 25.8 (C-2″), 18.1 (C-3″); HRMS-ESI *m/z* 535.1838 (M + H)^+^ (calculated for C_26_H_27_ClN_2_O_2_, 434.1764).

*3-{[(4-Fluorobenzyl)amino]methyl}-7-methoxy-1-(3-methylbut-2-en-1-yl)-9H-carbazol-2-ol* (**2d**). 16.64 mg; 30%; greenish yellow oil; purity HPLC-DAD 98.7%; mp: 94.2–95.1 °C; IR (KBr) *v_max_* cm^−1^: 3418, 2923, 1620, 1456, 1377, 1077, 1225, 803; ^1^H NMR (CDCl_3_, 300 MHz) δ: 7.84 (1H, br, NH), 7.75 (1H, d, *J* = 8.5 Hz, H-5), 7.45 (1H, s, H-4), 7.30 (dt, 2H, *J* = 9.4, 3.0 Hz, H-6”), 7.03 (2H, m, H-7”), 6.89 (1H, d, *J* = 2.2 Hz, H-8), 6.78 (1H, dd, *J* = 8.5, 2.2 Hz, H-6), 5.29 (1H, m, H-1”), 4.12 (2H, s, H-3′), 3.88 (3H, s, H-7′), 3.83 (2H, s, H-5′), 3.75 (s, 1H, NH), 3.64 (2H, d, *J* = 6.7 Hz, H-1′), 1.90 (3H, s, H-2″), 1.76 (3H, d, *J* = 1.1 Hz, H-3”); ^13^C NMR (CDCl_3_, 75 MHz): 160.7 (C-8′), 157.9 (C-7), 153.2 (C-2), 140.6 (C-8a), 140.0 (C-9a), 134.4 (C-6′), 132.9 (C-2′), 130.0 (C-6″), 122.5 (C-1″), 119.7 (C-5), 117.9 (C-5a), 116.9 (C-4), 115.7 (C-4a), 115.4 (C-7″), 115.1 (C-3), 109.8 (C-1), 107.7 (C-6), 95.0 (C-8), 55.7 (C-7′), 52.6 (C-3′), 51.8 (C-5′), 25.8 (C-2″), 23.8 (C-1′), 18.1 (C-3″); HRMS-ESI *m/z* 419.2127 (M + H)^+^ (calculated for C_26_H_27_FN_2_O_2_, 418.2050).

*3-{[(4-Bromobenzyl)amino]methyl}-7-methoxy-1-(3-methylbut-2-en-1-yl)-9H-carbazol-2-ol* (**2e**). 54.64 mg; 86%; greenish yellow oil; purity HPLC-DAD 98.1%; mp: 117.1–118.4 °C; IR (KBr) *v_max_* cm^−1^: 3445, 2919, 1652, 1449, 1361, 801, 669; ^1^H NMR (CDCl_3_, 300 MHz) δ: 7.85 (1H, br, NH), 7.75 (1H, d, *J* = 8.5 Hz, H-5), 7.48 (2H, m C-6”), 7.44 (1H, s, H-4), 7.21 (dd, 2H, *J* = 8.5, 2.5 Hz, H-6”), 6.89 (1H, d, *J* = 2.2 Hz, H-8), 6.78 (1H, dd, *J* = 8.5, 2.3 Hz, H-6), 5.35 (1H, m, H-1”), 4.11 (2H, s, H-3′), 3.88 (3H, s, H-7′), 3.81 (2H, s, H-5′), 3.65 (2H, d, *J* = 6.7 Hz, H-1′), 1.90 (3H, s, H-2″), 1.76 (3H, d, *J* = 1.1 Hz, H-3”); ^13^C NMR (CDCl_3_, 75 MHz): 157.9 (C-7), 153.2 (C-2), 140.6 (C-8a), 140.0 (C-9a), 137.5 (C-6′), 132.9 (C-2′), 131.8 (C-6”), 130.1 (C-7”), 122.6 (C-1”), 121.4 (C-8′), 119.7 (C-5), 117.8 (C-5a), 116.9 (C-4), 115.7 (C-4a), 115.3 (C-3), 109.8 (C-1), 107.8 (C-6), 95.0 (C-8), 55.7 (C-7′), 52.6 (C-3′), 51.8 (C-5′), 25.8 (C-2″), 23.8 (C-1′), 18.1 (C-3”); HRMS-ESI *m/z* 179.1329 (M + H)^+^ (calculated for C_26_H_27_BrN_2_O_2_, 478.1256).

*3-[(5-Amino-3,4-dihydroisoquinolin-2(1H)-yl)methyl]-7-methoxy-1-(3-methylbut-2-en-1-yl)-9H-carbazol-2-ol* (**2f**). 18.8 mg; 25.22%; orange solid; purity HPLC-DAD 98.9%; mp: 168.7–170.1 °C; IR (KBr) *v_max_* cm^−1^: 3387, 2919, 1617, 1449, 1361, 1257; ^1^H NMR (CDCl_3_, 300 MHz) δ: 7.86 (1H, br, NH), 7.76 (1H, d, *J* = 8.5 Hz, H-5), 7.49 (1H, s, H-4), 7.69 (1H, dd, *J* = 8.9 and 6.6 Hz, H-11′), 6.89 (1H, d, *J* = 2.2 Hz, H-8), 6.79 (1H, dd, *J* = 8.5 and 2.2 Hz, H-6), 6.57 (1H, d, *J* = 7.5 Hz, H-12′), 6.47 (1H, d, *J* = 7.5 Hz, H-10′), 5.35 (1H, ddd, *J* = 6.8, 5.4 and 1.4 Hz, H-1”), 4.00 (2H, s, H-3′), 3.89 (3H, s, H-7′), 3.57 (2H, s, H-6′), 3.60 (2H, d, *J* = 6.8 Hz, H-1′), 2.89 (2H, t, *J* = 6.0 Hz, H-5′), 2.61 (2H, t *J* = 7.7 Hz, H-4′), 1.88 (3H, s, H-2″), 1.75 (2H, d *J* = 1.1 Hz, H-3”); ^13^C NMR (CDCl_3_, 75 MHz): 157.8 (C-7), 154.1 (C-2), 144.1 (C-9′), 140.7(C-8a), 139.8 (C-9a), 136.3 (C-7′), 133.0 (C-2′), 126.4 (C-11′), 122.5 (C-1′), 119.8 (C-5), 119.5 (C-8′), 117.8 (C-5a), 117.5 (C-4), 117.1 (C-12′), 115.7 (C-4a), 115.4 (C-3), 112.7 (C-10′), 109.6 (C-1), 107.6 (C-6), 95.1 (C-8), 56.7 (C-6′), 55.4 (C-7”), 52.8 (C-3′), 51.0 (C-4′), 25.7 (C-2″), 24.9 (C-5′), 23.9 (C-1′), 18.1 (C-3”); HRMS-ESI *m/z* 440.2323 (M + H)^+^ (calculated for C_28_H_31_N_3_O_2_, 441.2416).

*3-{[(3-(Dimethylamino)propyl)(methyl)amino]methyl}-7-methoxy-9H-carbazol-2-ol* (**3a**) 23.27 mg; 51.38%; greenish yellow solid; purity HPLC-DAD 95.8%; mp: 148.2–150.0 °C; IR (KBr) *v_max_* cm^−1^: 3319, 2924, 1632, 1439, 1374, 1205, 740; ^1^H NMR (CDCl_3_, 300 MHz) δ: 7.99 (1H, br, H-9), 7.75 (1H, d, *J* = 8.5 Hz, H-5), 7.53 (1H, s, H-4), 6.99 (1H, s, H-2′), 6.87 (1H, d, *J* = 2.1 Hz, H-8), 6.83 (1H, s, H-1), 6.79 (1H, dd, *J* = 8.5, 2.3 Hz, H-6), 3.88 (3H, s, H-7′), 3.83 (2H, s, H-3′), 2.58 (2H, dd, *J* = 14.7 and 7.4 Hz, H-4′), 2.32 (3H, s, H-4”), 2.22 (6H, s, H-6”), 2.02 (2H, dd, *J* = 14.3 and 6.8 Hz, H-6′), 1.76 (2H, dt J 14.7 and 7.4, H-6′); ^13^C NMR (CDCl_3_, 75 MHz) 157.9 (C-7), 153.1 (C-2), 140.6 (C-8a), 139.9 (C-9a), 132.9 (C-2′), 122.7 (C-1″), 119.7 (C-5), 117.9 (C-5a), 116.9 (C-4), 115.6 (C-4a), 115.1 (C-3), 109.4 (C-1), 107.7 (C-6), 95.0 (C-8), 62.3 (C-3′), 57.1 (C-6′), 55.7 (C-4′), 44.8 (C-6″), 41.1 (C-4″), 25.9 (C-3″), 24.2 (C-5′), 18.1 (C-2″); HRMS-ESI *m/z* 342.2164 (M + H)^+^ (calculated for C_20_H_27_N_3_O_2_, 341.2103).

### 3.4. Crystallography

A single crystal was mounted on a cryoloop using paratone. X-ray diffraction data were collected at room temperature with a Gemini PX Ultra ((Rigaku/Oxford, Neu-Isenburg, Germany)) equipped with CuK_α_ radiation (λ = 1.54184 Å). The structure was solved by direct methods using SHELXS-97 [35] and refined with SHELXL-97 [35]. Crystal was monoclinic, space group P2_1_/c, cell volume 2047.89(15) Å^3^ and unit cell dimensions *a* = 18.0056(9) Å, *b* = 9.3592(3) Å, and *c* = 12.7126(6) Å and β = 107.074(5)° (uncertainties in parentheses). Non-hydrogen atoms were refined anisotropically. Hydrogen atoms were either placed at their idealized positions using appropriate HFIX instructions in SHELXL and included in subsequent refinement cycles or were directly found from difference Fourier maps and were refined freely with isotropic displacement parameters. The refinement converged to R (all data) = 12.46% and wR2 (all data) = 29.12%.

### 3.5. Yeast Screening Assay

*Saccharomyces cerevisiae* cells expressing human mutp53 R280K, Y220C, G245S, G245D, R273C, R273H, R175H, R248W, R248Q, and R282W (or empty vector as control) were obtained in previous works [36]. Yeast cells expressing human wtp53 were also obtained in previous work [37] and were used as positive controls. For expression of human wtp53 or mutp53, cells (routinely grown in minimal selective medium) were incubated in galactose selective medium with all the amino acids required for yeast growth (50 μg/mL) except leucine as described [36], in the presence of 10 μM of aminocarbazole derivatives, compounds **1** and **1b**–**1d**, or 0.1% DMSO, for approximately 42 h (time required by control yeast incubated with DMSO to achieve 0.4 OD_600_). Yeast growth was analyzed by colony-forming unit counts as described. Percentage of growth inhibition was calculated considering the wtp53-induced yeast growth inhibition as 100%.

### 3.6. Human Tumor Cell Lines and Growths Conditions

Human colon adenocarcinoma HCT116 cell lines expressing wt p53 were provided by B. Vogelstein (The Johns Hopkins Kimmel Cancer Center, Baltimore, MD, USA); human colon adenocarcinoma HT-29, breast adenocarcinoma MDA-MB-468, colon cancer SW837 and LS-1034, melanoma A375, glioblastoma SF-268, and non-tumorigenic foreskin fibroblasts HFF-1 cell lines were purchase from American Type Culture Collection (ATCC). Human hepatocarcinoma HuH-7 cell lines were purchase from JCRB cell bank. Tumor cells were routinely cultured in RPMI-1640 medium with UltraGlutamine (Lonza, VWR, Carnaxide, Portugal) supplemented with 10% fetal bovine serum (FBS; Gibco, Alfagene, Lisboa, Portugal). HFF-1 cells were cultured in DMEM/F-12 supplemented with 10% FBS. All cells were maintained at 37 °C in a humidified atmosphere of 5% CO_2_. Cells were routinely tested for mycoplasma infection using the MycoAlert™ PLUS mycoplasma detection kit (Lonza, VWR, Carnaxide, Portugal).

### 3.7. Sulforhodamine B (SRB) Assay

Human cell lines were seeded in 96-well plates at a density of 5.0 × 10^3^ (HCT116, HuH-7, A375, HT-29, SW837, MDA-MB-468, SF-268 and LS-1034), and 1.0 × 10^4^ (HFF-1) cells/well, and allowed to adhere for 24 h. Cells were treated with serial dilutions of compounds for additional 48 h. The effect on cell proliferation was measured by sulforhodamine B (SRB) assay, as described [37], and IC_50_ (concentration that causes 50% growth inhibition) values were determined for each cell line using the GraphPad Prism software (version 6.0, GraphPad, San Diego, CA, USA).

## 4. Conclusions

A series of new semi-synthetic aminocarbazoles derived from carbazoles natural products was successfully obtained and evaluated regarding the in vitro tumor growth inhibition activity and potential ability to activate p53 of the compounds. The results revealed a modest tumor growth inhibitory activity and no selectivity to the p53 pathway, in human tumor cells for the natural products heptaphylline (**1**), 7-methoxy-heptaphylline (**2**), and 7-methoxy-mukonal (**3**). Despite this, the results obtained indicate that aminocarbazole semi-synthetic derivatives, particularly 3-(*p*-fluoro)aminoheptaphylline (**1d**), may represent a promising starting point to search for new mutant p53-reactivating agents with promising application in cancer therapy.

## Figures and Tables

**Table 1:** Semi-synthesis of aminocarbazoles compounds **1a**–**1e**, **2a**–**2f**, and **3a** from natural-occurring carbazoles heptaphylline (**1**), 7-methoxy-heptaphylline (**2**), and 7-methoxy-mukonal (**3**). Sub. = substrate.

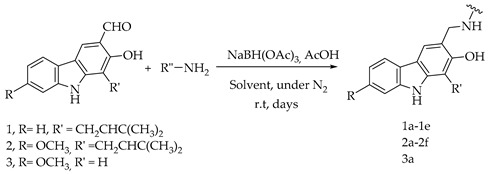						
Entry	Sub.	Amine Precursors	Products	Solvent	Time (Days)	Yield (%)
**1**	**1**	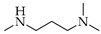	**1a**	THF DCE	5 3	39 49
**2**	**1**	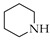	**1b**	THF DCE	4 3	44 90
**3**	**1**	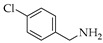	**1c**	THF DCE	3 -	31 -
**4**	**1**	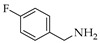	**1d**	THF DCE	3 3	15 42
**5**	**1**	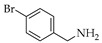	**1e**	THF DCE	5 3	47 64
**6**	**2**	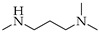	**2a**	THF DCE	5 4	16 51
**7**	**2**	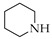	**2b**	THF DCE	8 5	21 39
**8**	**2**	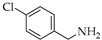	**2c**	THF DCE	4 4	15 34
**9**	**2**	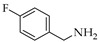	**2d**	THF DCE	4 4	13 30
**10**	**2**	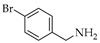	**2e**	THF DCE	5 3	35 86
**11**	**2**	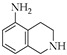	**2f**	THF	10	25
**12**	**3**	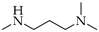	**3a**	THF	7	51

**Table 2:** Growth inhibition (GI_50_) concentration of **1**, **2**, **3**, and amino carbazoles **1a**–**1e** and **2a**–**2e** on human tumor cell lines.

Cell Line	HCT116 (wt)	HT-29 (R273H)	HuH-7 (Y220C)	SW837 (R248W)	MDA-MB-468 (R273H)	A375	SF-268 (R273H)	LS-1034 (G245S)
**1**	4.4 ± 1.1	14.0 ± 0.1	6.1 ± 0.3	7.2 ± 0.4	<3.13	-	-	-
**2**	15.0 ± 1.0	23.0 ± 6.0	21.5 ± 0.5	30.5 ± 2.5	-	5.0 ± 0.3	-	-
**3**	4.7 ± 0.5	9.4 ± 0.7	6.3± 2.2	6.1 ± 1.2	-	-	-	-
**1a**	> 50	>50	-	-	>50	-	-	-
**1b**	18.1 ± 0.9	18.0 ± 1.9	> 50	23.0 ± 3.0	16.0 ± 2.0	-	-	-
**1c**	22.6 ± 4.5	-	30.5 ± 0.5	29.0 ± 2.0	-	-	-	-
**1d**	15.5 ± 1.5	8.3 ± 1.0	28.5 ± 0.5	26.0 ± 2.0	18.0 ± 3.0	24.5 ± 1.5	20.0 ± 4.0	5.8 ± 0.8
**1e**	30.6 ± 2.8	-	>50	23.0 ± 1.0	-	38.2 ± 1.9	-	-
**1f**	-	-	-	-	-	-	-	-
**2a**	22.3 ± 1.0	-	15.5 ± 3.5	18.0 ± 0.0	-	29.7 ± 1.8	-	-
**2b**	24.0 ± 4.0	28.5 ± 1.5	>50	>50	-	>50	-	-
**2c**	25.0 ± 1.8	-	45.0 ± 3.0	>50	-	30.7 ± 3.7	-	-
**2d**	16.1 ± 3.5	-	23.0 ± 3.0	10.8 ± 1.3	-	27.8 ± 3.0	-	-
**2e**	27.0 ± 4.4	-	33.5 ± 5.5	37.5 ± 0.5	-	38.3 ± 3.2	-	-
**2f**	-	-	-	-	-	-	-	-
**2g**	-	-	-	-	-	-	-	-
**2h**	-	-	-	-	-	-	-	-
**3a**	22.2± 0.9	-	15.5 ± 3.5	18.0 ± 0.0	-	29.7 ± 1.8	-	-
**Etoposide**	0.54 ± 0.1	1.52 ± 0.3	3.66 ± 0.7	0.9 ± 0.07	2.07 ± 0.15	0.85 ± 0.09	-	-

Concentration that induces 50% of growth inhibition (IC_50_) was determined by the sulforhodamine B (SRB) assay after 48 h treatment. Data are mean ± standard error of the mean (SEM) of 3–4 independent experiments. Dash means not detected.

**Table 3:** Effect of heptaphylline and amine derivatives **1b**–**1d** on the growth of yeast cells expressing wild-type (wt) or mutant p53.

Mutant p53	1	1b	1c	1d
R280K	-	-	-	-
Y220C	71.17 ± 11.53	-	-	47.83 ± 5.80
G245D	-	57.06 ± 13.03	34.73 ± 12.50	63.43 ± 11.50
R273H	-	-	-	-
R175H	-	-	41.50 ± 16.40	-
R248W	-	-	-	34.30 ± 2.97
R248Q	-	-	52.90 ± 9.13	46.20 ± 5.27
R273C	-	47.30 ± 6.07	-	-
R282W	-	42.33 ± 1.63	83.63 ± 7.16	-
G245S	68.40 ± 12.20	47.87 ± 8.83	52.20 ± 6.90	55.33 ± 4.53
wt p53	-	61.70 ± 11.1	-	-

## Data Availability

The data presented in this study are available in this article and respective supplementary information.

## Supplementary Materials

The following are available online, Figure S1. ^1^H NMR spectrum of *3-{[(3-(Dimethylamino)propyl)(methyl)amino]methyl}-1-(3-methylbut-2-en-1-yl)-9H-carbazol-2-ol* (**1a**). (CDCl_3_, 300, MHz). Figure S2. ^13^C NMR spectrum of *3-{[(3-(Dimethylamino)propyl)(methyl)amino]methyl}-1-(3-methylbut-2-en-1-yl)-9H-carbazol-2-ol* (**1a**). (CDCl_3_, 75, MHz). Figure S3. HMBC spectrum of *3-{[(3-(Dimethylamino) propyl)(methyl)amino]methyl}-1-(3-methylbut-2-en-1-yl)-9H-carbazol-2-ol* (**1a**). (CDCl_3_, 300 MHz). Figure S4. ^1^H NMR spectrum *1-(3-Methylbut-2-en-1-yl)-3-(piperidin-1-ylmethyl)-9H-carbazol-2-ol* (**1b**) (CDCl_3_, 300, MHz). Figure S5. ^13^C NMR spectrum of *1-(3-Methylbut-2-en-1-yl)-3-(piperidin-1-ylmethyl)-9H-carbazol-2-ol* (**1b**) (CDCl_3_, 75, MHz). Figure S6. HMBC spectrum of *1-(3-Methylbut-2-en-1-yl)-3-(piperidin-1-ylmethyl)-9H-carbazol-2-ol* (**1b**) (CDCl_3_, 300 MHz). Figure S7. ^1^H NMR spectrum of *3-{[(4-Chlorobenzyl)amino]methyl}-1-(3-methylbut-2-en-1-yl)-9H-carbazol-2-ol* (**1c**) (CDCl_3_, 300, MHz). Figure S8. ^13^C NMR spectrum of *3-{[(4-Chlorobenzyl)amino]methyl}-1-(3-methylbut-2-en-1-yl)-9H-carbazol-2-ol* (**1c**) (CDCl_3_, 75, MHz). Figure S9. ^1^H NMR spectrum of *3-{[(4-Fluorobenzyl)amino]methyl}-1-(3-methylbut-2-en-1-yl)-9H-carbazol-2-ol* (CDCl_3_, 300, MHz). Figure S10. ^13^C NMR spectrum of *3-{[(4-Fluorobenzyl)amino]methyl}-1-(3-methylbut-2-en-1-yl)-9H-carbazol-2-ol* (**1d**) (CDCl_3_, 75, MHz). Figure S11: ^1^H NMR spectrum of *3-{[(4-Bromobenzyl)amino]methyl}-1-(3-methylbut-2-en-1-yl)-9H-carbazol-2-ol* (**1e**) (CDCl_3_, 300, MHz). Figure S12: ^13^C NMR spectrum of *3-{[(4-Bromobenzyl)amino]methyl}-1-(3-methylbut-2-en-1-yl)-9H-carbazol-2-ol* (**1e**) (CDCl_3_, 75, MHz). Figure S13: HMBC spectrum of *3-{[(4-Bromobenzyl)amino]methyl}-1-(3-methylbut-2-en-1-yl)-9H-carbazol-2-ol* (**1e**) (CDCl_3_, 300, MHz). Figure S14: ^1^H NMR spectrum of *3-{[(3-(Dimethylamino)propyl)(methyl) amino]methyl}-7-methoxy-1-(3-methylbut-2-en-1-yl)-9H-carbazol-2-ol* (**2a**) CDCl_3_, 300, MHz). Figure S15: ^13^C NMR spectrum of *3-{[(3-(Dimethylamino)propyl)(methyl)amino]methyl}-7-methoxy-1-(3-methylbut-2-en-1-yl)-9H-carbazol-2-ol* (**2a**) (CDCl_3_, 75, MHz). Figure S16: HRMS spectrum of *3-{[(3-(Dimethylamino)propyl)(methyl)amino]methyl}-7-methoxy-1-(3-methylbut-2-en-1-yl)-9H-carbazol-2-ol* (**2a**) (CDCl_3_, 300, MHz). Figure S17: ^1^H NMR spectrum of *7-Methoxy-1-(3-methylbut-2-en-1-yl)-3-(piperidin-1-ylmethyl)-9H-carbazol-2-ol* (**2b**). (CDCl_3_, 300, MHz). Figure S18: ^13^C NMR spectrum of *7-Methoxy-1-(3-methylbut-2-en-1-yl)-3-(piperidin-1-ylmethyl)-9H-carbazol-2-ol* (**2b**). (CDCl_3_, 75, MHz). Figure S19: HMBC spectrum of *7-Methoxy-1-(3-methylbut-2-en-1-yl)-3-(piperidin-1-ylmethyl)-9H-carbazol-2-ol* (**2b**). (CDCl_3_, 300, MHz). Figure S20: ^1^H NMR spectrum of *3-{[(4-Chlorobenzyl)amino]methyl}-7-methoxy-1-(3-methylbut-2-en-1-yl)-9H-carbazol-2-ol* (**2c**)*,* (CDCl_3_, 300, MHz). Figure S21: ^13^C NMR spectrum of *3-{[(4-Chlorobenzyl)amino]methyl}-7-methoxy-1-(3-methylbut-2-en-1-yl)-9H-carbazol-2-ol* (**2c**) (CDCl_3_, 75, MHz). Figure S22: HMBC spectrum of *3-{[(4-Chlorobenzyl)amino]methyl}-7-methoxy-1-(3-methylbut-2-en-1-yl)-9H-carbazol-2-ol* (**2c**) (CDCl_3_, 300, MHz). Figure S23: ^1^H NMR spectrum of *3-{[(4-Fluorobenzyl)amino]methyl}-7-methoxy-1-(3-methylbut-2-en-1-yl)-9H-carbazol-2-ol* (**2d**) (CDCl_3_, 300, MHz). Figure S24: ^13^C NMR spectrum of *3-{[(4-Fluorobenzyl)amino]methyl}-7-methoxy-1-(3-methylbut-2-en-1-yl)-9H-carbazol-2-ol* (**2d**) (CDCl_3_, 75, MHz). Figure S25: HMBC spectrum of *3-{[(4-Fluorobenzyl)amino]methyl}-7-methoxy-1-(3-methylbut-2-en-1-yl)-9H-carbazol-2-ol* (**2d**) (CDCl_3_, 300, MHz). Figure S26: ^1^H NMR spectrum *3-{[(4-Bromobenzyl)amino]methyl}-7-methoxy-1-(3-methylbut-2-en-1-yl)-9H-carbazol-2-ol* (**2e**) (CDCl_3_, 300, MHz). Figure S27: ^13^C NMR spectrum of *3-{[(4-Bromobenzyl)amino]methyl}-7-methoxy-1-(3-methylbut-2-en-1-yl)-9H-carbazol-2-ol* (**2e**) (CDCl_3_, 75, MHz). Figure S28: ^13^C NMR spectrum of *3-{[(4-Bromobenzyl)amino]methyl}-7-methoxy-1-(3-methylbut-2-en-1-yl)-9H-carbazol-2-ol* (**2e**) (CDCl_3_, 300, MHz). Figure S29: ^1^H NMR spectrum of *3-[(5-Amino-3,4-dihydroisoquinolin-2(1H)-yl)methyl]-7-methoxy-1-(3-methylbut-2-en-1-yl)-9H-carbazol-2-ol* (**2f**). (CDCl_3_, 300, MHz). Figure S30: ^13^C NMR spectrum of *3-[(5-Amino-3,4-dihydroisoquinolin-2(1H)-yl)methyl]-7-methoxy-1-(3-methylbut-2-en-1-yl)-9H-carbazol-2-ol* (**2f**). (CDCl_3_, 75, MHz). Figure S31: ^1^H NMR spectrum of *3-{[(3-(Dimethylamino)propyl)(methyl)amino]methyl}-7-methoxy-9H-carbazol-2-ol* (**3a**) (CDCl_3_, 300, MHz). Figure S32: ^13^C NMR spectrum of *3-{[(3-(Dimethylamino)propyl)(methyl)amino]methyl}-7-methoxy-9H-carbazol-2-ol* (**3a**) (CDCl_3_, 75, MHz). Figure S33: HRMS of compound *3-{[(3-(Dimethylamino)propyl)(methyl)amino] methyl}-1-(3-methylbut-2-en-1-yl)-9H-carbazol-2-ol* (**1a**), (20, 300 V). Figure S34: HRMS of compound *1-(3-Methylbut-2-en-1-yl)-3-(piperidin-1-ylmethyl)-9H-carbazol-2-ol* (**1b**), (20, 300 V). Figure S35: HRMS of compound of compound *3-{[(4-Chlorobenzyl)amino]methyl}-1-(3-methylbut-2-en-1-yl)-9H-carbazol-2-ol* (**1c**), (20, 300 V). Figure S36: HRMS of compound *3-{[(4-Fluorobenzyl)amino]methyl}-1-(3-methylbut-2-en-1-yl)-9H-carbazol-2-ol* (**1d**) (20, 300 V). Figure S37: HRMS of compound *3-{[(4-Bromobenzyl)amino]methyl}-1-(3-methylbut-2-en-1-yl)-9H-carbazol-2-ol* (**1e**). (20, 300V). Figure S38: HRMS of compound *3-{[(3-(Dimethylamino) propyl)(methyl)amino]methyl}-7-methoxy-1-(3-methylbut-2-en-1-yl)-9H-carbazol-2-ol* (**2a**), (20, 300 V). Figure S39: HRMS of compound *3-{[(4-Chlorobenzyl)amino]methyl}-7-methoxy-1-(3-methylbut-2-en-1-yl)-9H-carbazol-2-ol* (**2c**), (20, 300 V). Figure S40: HRMS of compound *3-{[(4-Fluorobenzyl)amino]methyl}-7-methoxy-1-(3-methylbut-2-en-1-yl)-9H-carbazol-2-ol* (**2d**), (20, 300 V). Figure S41: HRMS of compound *3-{[(4-Bromobenzyl)amino]methyl}-7-methoxy-1-(3-methylbut-2-en-1-yl)-9H-carbazol-2-ol* (**2e**), (20, 300 V). Figure S42: HRMS of compound *3-[(5-Amino-3,4-dihydroisoquinolin-2(1H)-yl)methyl]-7-methoxy-1-(3-methylbut-2-en-1-yl)-9H-carbazol-2-ol* (**2f**), (20, 300 V). Figure S43: HRMS of compound *3-{[(3-(Dimethylamino)propyl)(methyl)amino]methyl}-7-methoxy-9H-carbazol-2-ol* (**3a**)*,* (20, 300 V).

## Sample Availability

Samples of the compounds **1**–**3** and derivatives are available from the authors.

## References

[1] Xin Z.-Q., Lu J.-J., Ke C.-Q., Hu C.-X., Lin L.-P., Ye Y. (2008). Constituents from Clausena excavata. Chem. Pharm. Bull..

[2] Knölker H.-J., Reddy K.R. (2002). Isolation and Synthesis of Biologically Active Carbazole Alkaloids. Chem. Rev..

[3] Wu T.-S., Huang S.-C., Wu P.-L., Kuoh C.-S. (1999). Alkaloidal and other constituents from the root bark of Clausena excavata. Phytochemistry.

[4] Das K.C., Chakraborty D.P., Bose P.K. (1965). Antifungal activity of some constituents ofMurraya koenigii spreng. Cell. Mol. Life Sci..

[5] Chakthong S., Bindulem N., Raknai S., Yodwaree S., Kaewsanee S., Kanjana-Opas A. (2016). Carbazole-pyranocoumarin conjugate and two carbazole alkaloids from the stems of Clausena excavata. Nat. Prod. Res..

[6] Thongthoom T., Promsuwan P., Yenjai C. (2011). Synthesis and cytotoxic activity of the heptaphylline and 7-methoxyheptaphylline series. Eur. J. Med. Chem..

[7] Thongthoom T., Songsiang U., Phaosiri C., Yenjai C. (2010). Biological activity of chemical constituents from Clausena harmandiana. Arch. Pharmacal Res..

[8] Yenjai C., Sripontan S., Sriprajun P., Kittakoop P., Jintasirikul A., Tanticharoen M., Thebtaranonth Y. (2000). Coumarins and Carbazoles with Antiplasmodial Activity from Clausena harmandiana. Planta Medica.

[9] Wu T.-S., Huang S.-C., Wu P.-L., Teng C.-M. (1996). Carbazole alkaloids from Clausena excavata and their biological activity. Phytochemistry.

[10] Maneerat W., Phakhodee W., Ritthiwigrom T., Cheenpracha S., Promgool T., Yossathera K., Deachathai S., Laphookhieo S. (2012). Antibacterial carbazole alkaloids from Clausena harmandiana twigs. Fitoterapia.

[11] Chakraborty A., Saha C., Podder G., Chowdhury B., Bhattacharyya P. (1995). Carbazole alkaloid with antimicrobial activity from clausena heptaphylla. Phytochemistry.

[12] Patel O.P.S., Mishra A., Maurya R., Saini D., Pandey J., Taneja I., Raju K.S.R., Kanojiya S., Shukla S.K., Srivastava M.N. (2016). Naturally Occurring Carbazole Alkaloids fromMurraya koenigiias Potential Antidiabetic Agents. J. Nat. Prod..

[13] Boonyarat C., Yenjai C., Vajragupta O., Waiwut P. (2015). Heptaphylline induces apoptosis in human colon adenocarcinoma cells through bid and Akt/NF-?B (p65) pathways. Asian Pac. J. Cancer Prev..

[14] Saturnino C., Iacopetta D., Sinicropi M.S., Rosano C., Caruso A., Caporale A., Marra N., Marengo B., Pronzato M.A., Parisi O.I. (2014). N-Alkyl Carbazole Derivatives as New Tools for Alzheimer’s Disease: Preliminary Studies. Molecules.

[15] Krahl M.P., Jäger A., Krause T., Knölker H.-J. (2006). First total synthesis of the 7-oxygenated carbazole alkaloids clauszoline-K, 3-formyl-7-hydroxycarbazole, clausine M, clausine N and the anti-HIV active siamenol using a highly efficient palladium-catalyzed approach. Org. Biomol. Chem..

[16] Wangboonskul J.D., Pummangura S., Chaichantipyuth C. (1984). Five Coumarins and a Carbazole Alkaloids From the Root Bark of Clausena harmandiana. J. Nat. Prod..

[17] Issa S., Walchshofer N., Kassab I., Termoss H., Chamat S., Geahchan A., Bouaziz Z. (2010). Synthesis and antiproliferative activity of oxazinocarbazole and *N*,*N*-bis(carbazolylmethyl)amine derivatives. Eur. J. Med. Chem..

[18] Chen Y.-L., Hung H.-M., Lu C.-M., Li K.-C., Tzeng C.-C. (2004). Synthesis and anticancer evaluation of certain indolo[2,3-b]quinoline derivatives. Bioorg. Med. Chem..

[19] Compain-Batissou M., Latreche D., Gentili J., Walchshofer N., Bouaziz Z. (2004). Synthesis and Diels–Alder Reactivity of ortho-Carbazolequinones. Chem. Pharm. Bull..

[20] Joerger A.C., Fersht A.R. (2007). Structural Biology of the Tumor Suppressor p53 and Cancer-Associated Mutants. Advances in Cancer Research.

[21] Vousden K.H., Lane D.P. (2007). p53 in health and disease. Nat. Rev. Mol. Cell Biol..

[22] Fridman J.S., Lowe S.W. (2003). Control of apoptosis by p53. Oncogene.

[23] Muller P.A., Vousden K.H. (2014). Mutant p53 in Cancer: New Functions and Therapeutic Opportunities. Cancer Cell.

[24] Yu X., Blanden A.R., Narayanan S., Jayakumar L., Lubin D., Augeri D., Kimball S.D., Loh S.N., Carpizo D.R. (2014). Small molecule restoration of wildtype structure and function of mutant p53 using a novel zinc-metallochaperone based mechanism. Oncotarget.

[25] Liu X., Wilcken R., Joerger A.C., Chuckowree I.S., Amin J., Spencer J., Fersht A.R. (2013). Small molecule induced reactivation of mutant p53 in cancer cells. Nucleic Acids Res..

[26] Boeckler F.M., Joerger A.C., Jaggi G., Rutherford T.J., Veprintsev D.B., Fersht A.R. (2008). Targeted rescue of a destabilized mutant of p53 by an in silico screened drug. Proc. Natl. Acad. Sci. USA.

[27] Bauer M.R., Jones R.N., Baud M.G.J., Wilcken R., Boeckler F.M., Fersht A.R., Joerger A.C., Spencer J. (2016). Harnessing Fluorine–Sulfur Contacts and Multipolar Interactions for the Design of p53 Mutant Y220C Rescue Drugs. ACS Chem. Biol..

[28] Rauf S.M.A., Endou A., Takaba H., Miyamoto A. (2013). Effect of Y220C Mutation on p53 and Its Rescue Mechanism: A Computer Chemistry Approach. Protein J..

[29] Roughley S.D., Jordan A.M. (2011). The Medicinal Chemist’s Toolbox: An Analysis of Reactions Used in the Pursuit of Drug Candidates. J. Med. Chem..

[30] Abdel-Magid A.F., Carson K.G., Harris B.D., Maryanoff C.A., Shah R.D. (1996). Reductive Amination of Aldehydes and Ketones with Sodium Triacetoxyborohydride. Studies on Direct and Indirect Reductive Amination Procedures1. J. Org. Chem..

[31] Joshi B., Kamat V., Gawad D., Govindachari T. (1972). Structure and synthesis of heptaphylline. Phytochemistry.

[32] Joshi B., Kamat V., Saksena A., Govindachari T. (1967). Structure of heptaphylline, a carbazole alkaloid from clausena heptaphylla wt. & arn. Tetrahedron Lett..

[33] Leão M., Moreira S., Soares J., Bessa C., Maciel C., Ciribilli Y., Pereira C., Inga A., Saraiva L. (2013). Novel simplified yeast-based assays of regulators of p53-MDMX interaction and p53 transcriptional activity. FEBS J..

[34] Jantamat P., Weerapreeyakul N., Puthongking P. (2019). Cytotoxicity and Apoptosis Induction of Coumarins and Carbazole Alkaloids from Clausena harmandiana. Molecules.

[35] Sheldrick G. (2008). A short history ofSHELX. Acta Crystallogr. Sect. A Found. Crystallogr..

[36] Soares J., Raimundo L., Pereira N.A., Monteiro Â., Gomes S., Bessa C., Pereira C., Queiroz G., Bisio A., Fernandes J. (2016). Reactivation of wild-type and mutant p53 by tryptophanolderived oxazoloisoindolinone SLMP53-1, a novel anticancer small-molecule. Oncotarget.

[37] Soares J., Raimundo L., Pereira N.A., dos Santos D.J., Pérez M., Queiroz G., Leão M., Santos M.M., Saraiva L. (2015). A tryptophanol-derived oxazolopiperidone lactam is cytotoxic against tumors via inhibition of p53 interaction with murine double minute proteins. Pharmacol. Res..

