# Supplementary material for: Semi-Synthesis of Small Molecules of Aminocarbazoles: Tumor Growth Inhibition and Potential Impact on p53
Source: Molecules. 2021 Mar 15;26(6):1637. doi: 10.3390/molecules26061637 (PMC7998292; doi:10.3390/molecules26061637)
Supplement: Supplementary file 1 [file molecules-26-01637-s001.zip › molecules-1126816-supplementary.pdf]

Supplementary information

Semi-Synthesis of Small Molecules of Aminocarbazoles: Tumor Growth Inhibition and Potential Impact on p53

Solida Long [1], Joana B. Loureiro [2], Carla Carvalho [2], Luís Gales [3,4], Lucília Saraiva [2,*], Madalena M. M. Pinto [1,5], Ploenthip Puthongking [6] and Emília Sousa [1,5,*]

[1] Laboratory of Organic and Pharmaceutical Chemistry (LQOF), Department of Chemical Sciences, Faculty of Pharmacy, University of Porto, Rua de Jorge Viterbo Ferreira, 228, 4050-313 Porto, Portugal; solidachhann@gmail.com (S.L.); madalena@ff.up.pt (M.M.M.P.)

[2] Laboratory of Microbiology (LAQV/REQUIMTE), Department of Biological Sciences, Faculty of Pharmacy, University of Porto, Rua de Jorge Viterbo Ferreira, 228, 4050-313 Porto, Portugal; up201407524@ff.up.pt (J.B.L.); pg32852@alunos.uminho.pt (C.C.)

[3] Institute for the Biomedical Science Abel Salazar (ICBAS), University of Porto, Rua de Jorge Viterbo Ferreira, 228, 4050-313 Porto, Portugal; lgales@ibmc.up.pt

[4] Instituto de Biologia Molecular e Celular (i3S-IBMC), University of Porto, Rua de Jorge Viterbo Ferreira, 228, 4050-313 Porto, Portugal

[5] Interdisciplinary Centre of Marine and Environmental Research (CIIMAR), 4450-208 Matosinhos, Portugal

[6] Department of Pharmaceutical Chemistry, Faculty of Pharmaceutical Sciences, Khon Kean University, Khon Kean, 40002, Thailand; pploenthip@kku.ac.th

* Correspondences: luciliasaraiva@ff.up.pt (L.S.); esousa@ff.up.pt (E.S.); Tel.: +351-2-2042-8689 (E.S.)

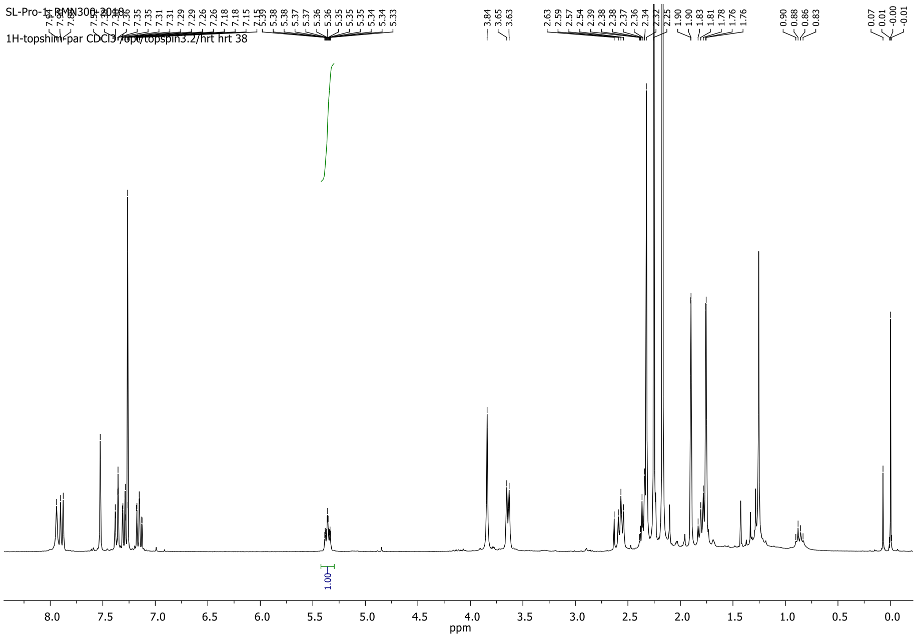

Figure S1. ^{1}H NMR spectrum of *3-{[(3-(Dimethylamino)propyl)(methyl)amino]methyl}-1-(3-methylbut-2-en-1-yl)-9H-carbazol-2-ol* (***1a***). ($CDCl_3$, 300, MHz).

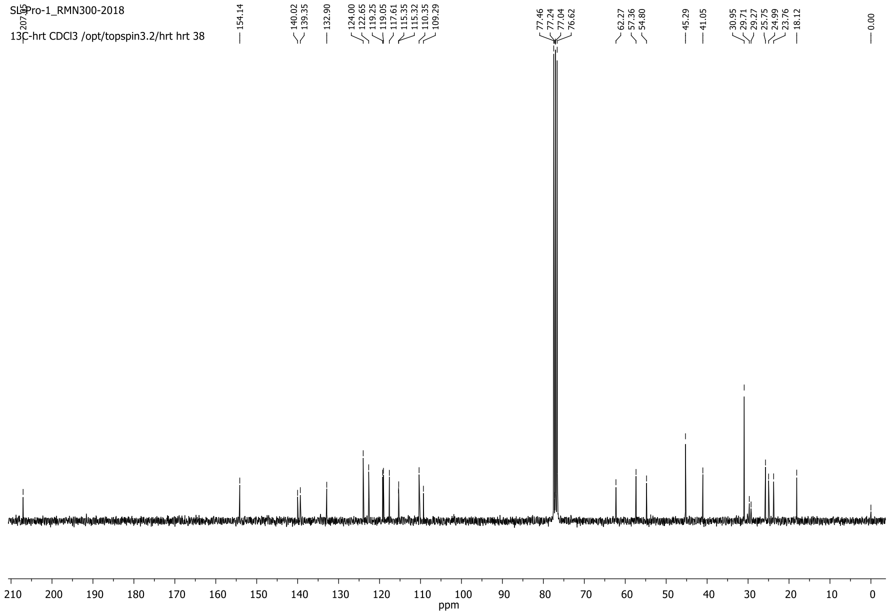

Figure S2. ^{13}C NMR spectrum of *3-{[(3-(Dimethylamino)propyl)(methyl)amino]methyl}-1-(3-methylbut-2-en-1-yl)-9H-carbazol-2-ol* (***1a***). ($CDCl_3$, 75, MHz).

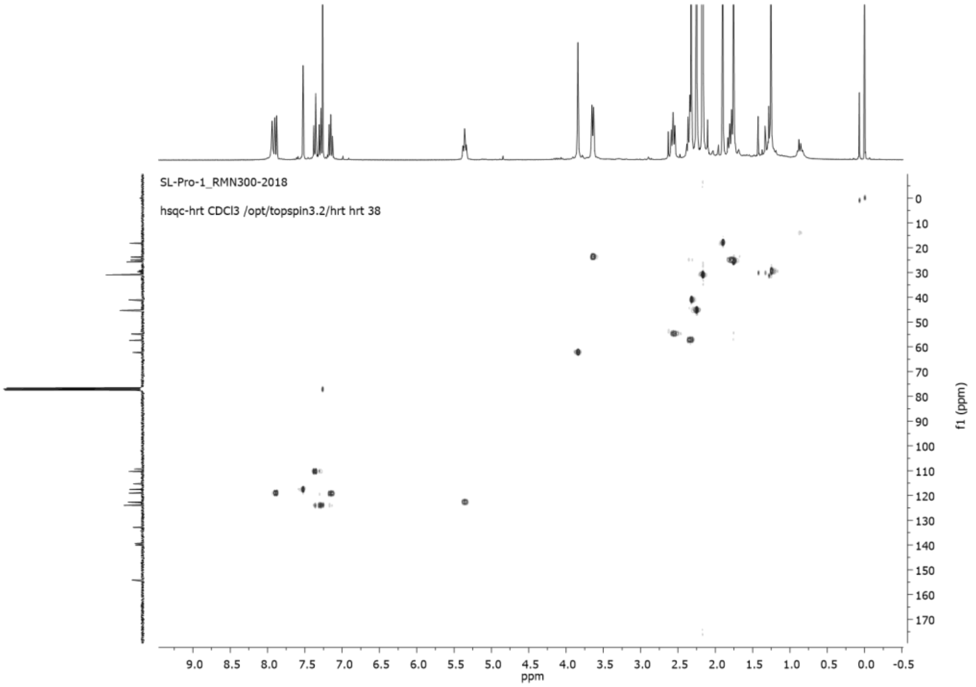

Figure S3. HMBC spectrum of *3-{[(3-(Dimethylamino)propyl)(methyl)amino]methyl}-1-(3-methylbut-2-en-1-yl)-9H-carbazol-2-ol* (***1a***). ($CDCl_3$, 300 MHz).

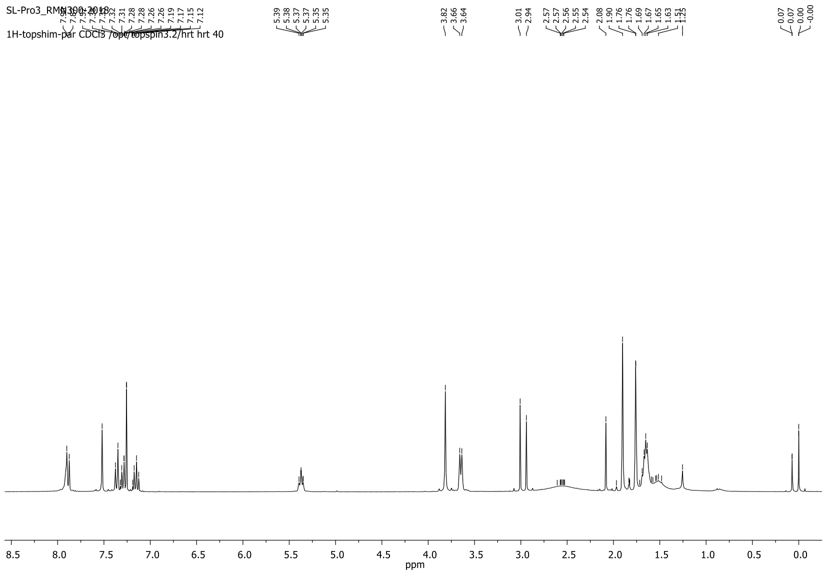

Figure S4. ^1H NMR spectrum *1-(3-Methylbut-2-en-1-yl)-3-(piperidin-1-ylmethyl)-9H-carbazol-2-ol (**1b**)* ($CDCl_3$, 300, MHz).

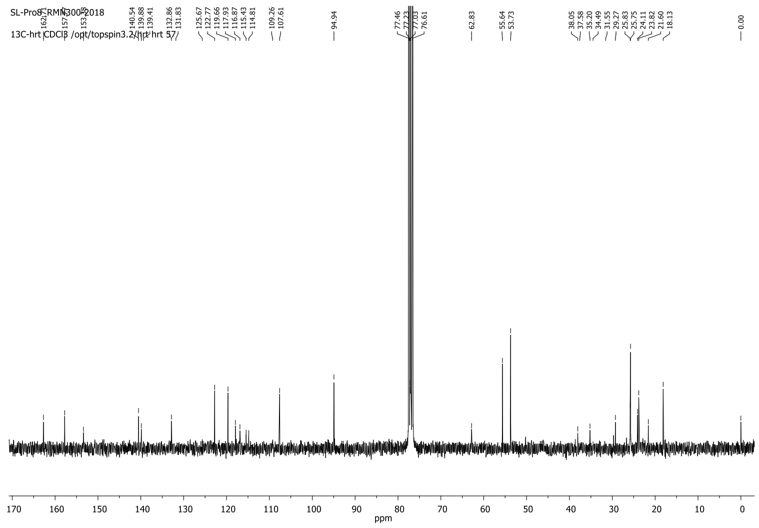

Figure S5. ^{13}C NMR spectrum of *1-(3-Methylbut-2-en-1-yl)-3-(piperidin-1-ylmethyl)-9H-carbazol-2-ol (**1b**)* ($CDCl_3$, 75, MHz).

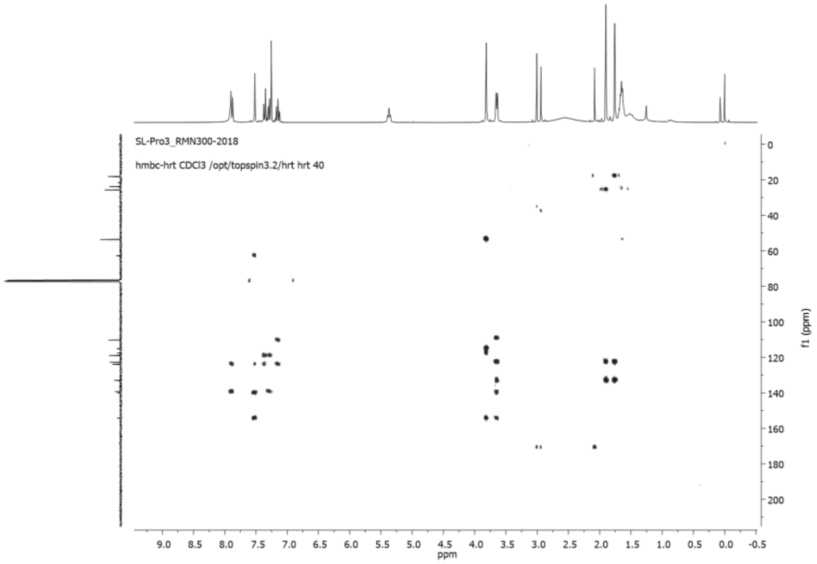

Figure S6. HMBC spectrum of *1-(3-Methylbut-2-en-1-yl)-3-(piperidin-1-ylmethyl)-9H-carbazol-2-ol (**1b**)* ($CDCl_3$, 300 MHz).

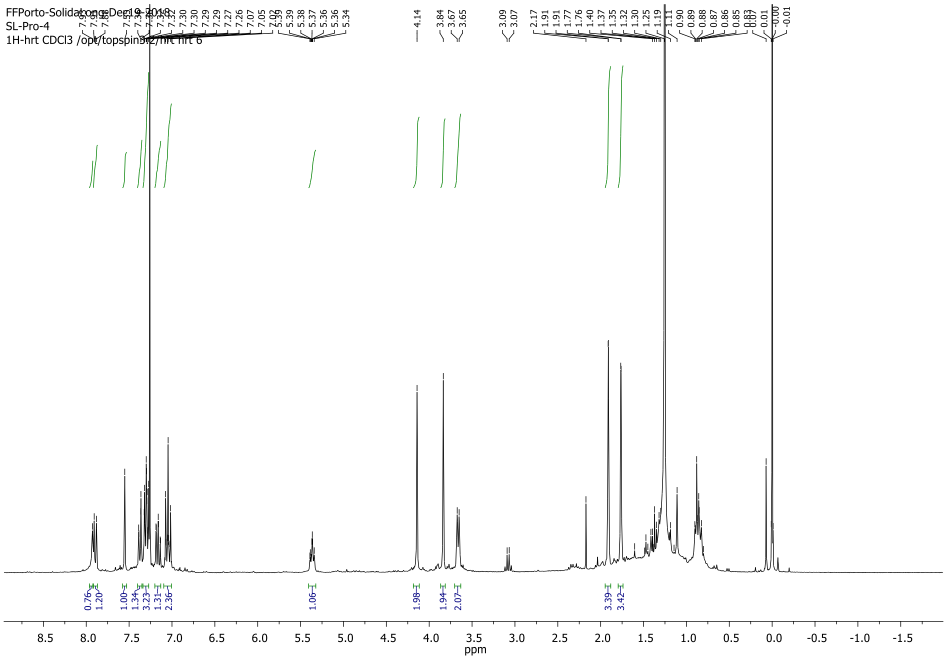

Figure S7. ^{1}H NMR spectrum of *3-{[(4-Chlorobenzyl)amino]methyl}-1-(3-methylbut-2-en-1-yl)-9H-carbazol-2-ol (**1c**)* ($CDCl_3$, 300, MHz).

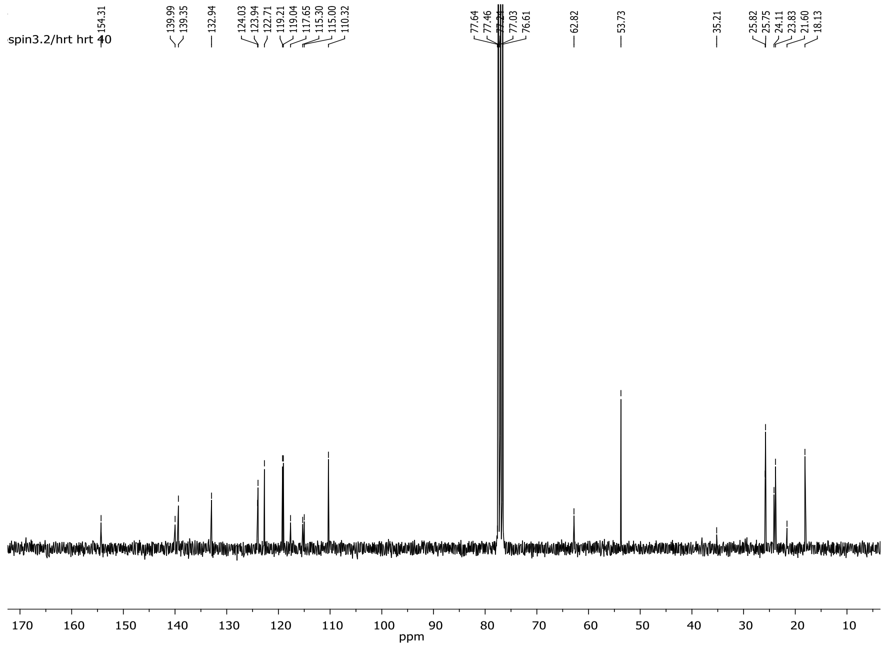

Figure S8. ^{13}C NMR spectrum of *3-{[(4-Chlorobenzyl)amino]methyl}-1-(3-methylbut-2-en-1-yl)-9H-carbazol-2-ol (**1c**)* ($CDCl_3$, 75, MHz).

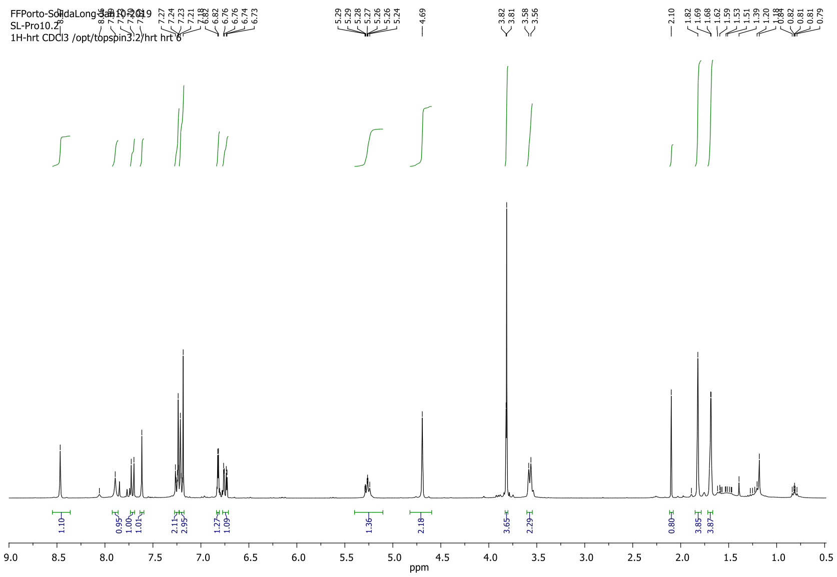

Figure S9. ^{1}H NMR spectrum of *3-{[(4-Fluorobenzyl)amino]methyl}-1-(3-methylbut-2-en-1-yl)-9H-carbazol-2-ol* ($CDCl_3$, 300, MHz).

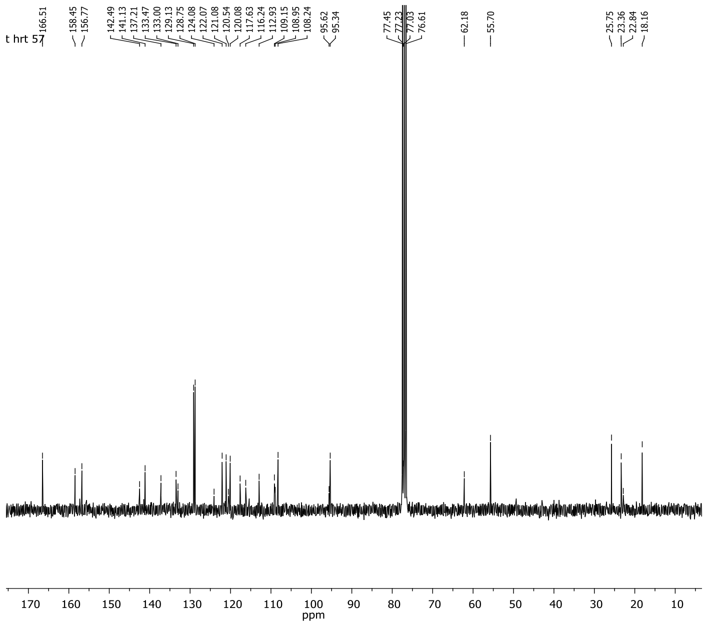

Figure S10. ^{13}C NMR spectrum of *3-{[(4-Fluorobenzyl)amino]methyl}-1-(3-methylbut-2-en-1-yl)-9H-carbazol-2-ol* ***(1d)*** ($CDCl_3$, 75, MHz).

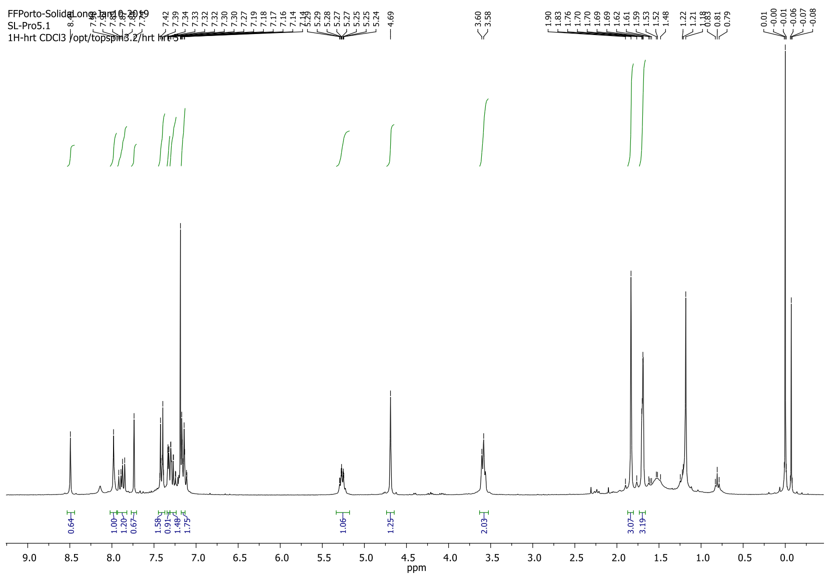

Figure S11: ^{1}H NMR spectrum of *3-{[(4-Bromobenzyl)amino]methyl}-1-(3-methylbut-2-en-1-yl)-9H-carbazol-2-ol (**1e**)* ($CDCl_3$, 300, MHz).

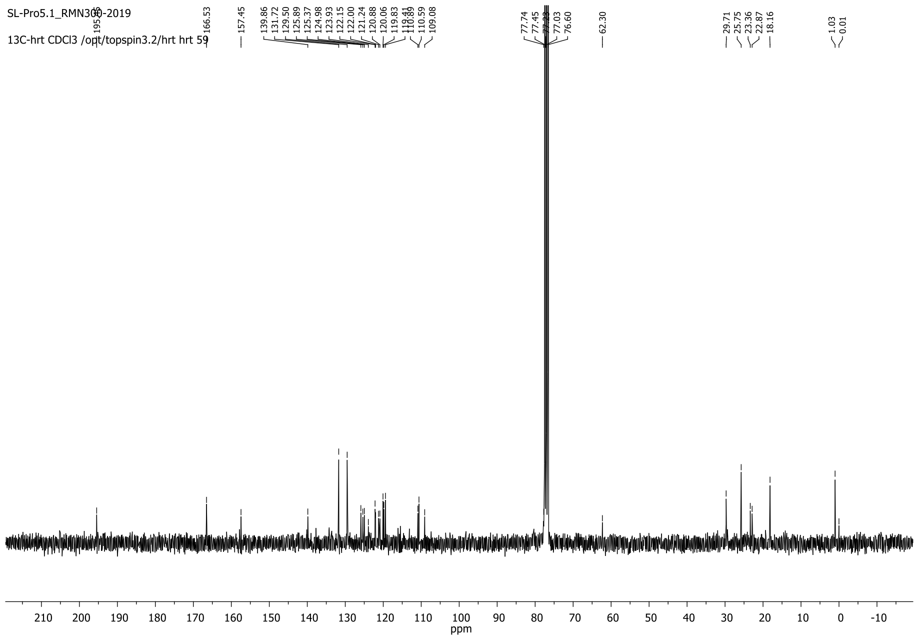

Figure S12: ^{13}C NMR spectrum of *3-{[(4-Bromobenzyl)amino]methyl}-1-(3-methylbut-2-en-1-yl)-9H-carbazol-2-ol (**1e**)* ($CDCl_3$, 75, MHz).

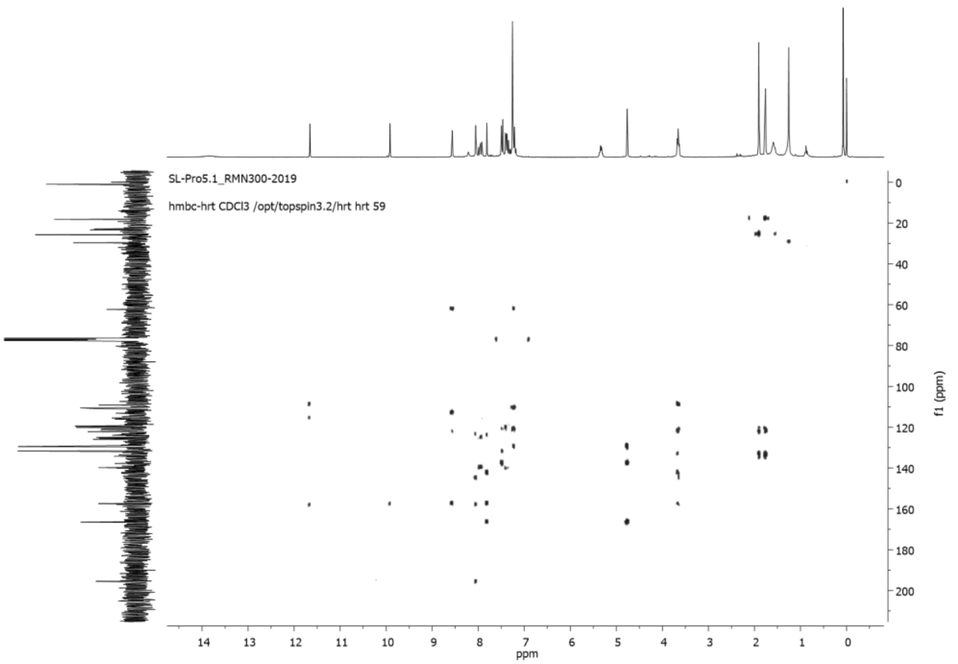

Figure S13: HMBC spectrum of *3-{[(4-Bromobenzyl)amino]methyl}-1-(3-methylbut-2-en-1-yl)-9H-carbazol-2-ol (**1e**)* ($CDCl_3$, 300, MHz).

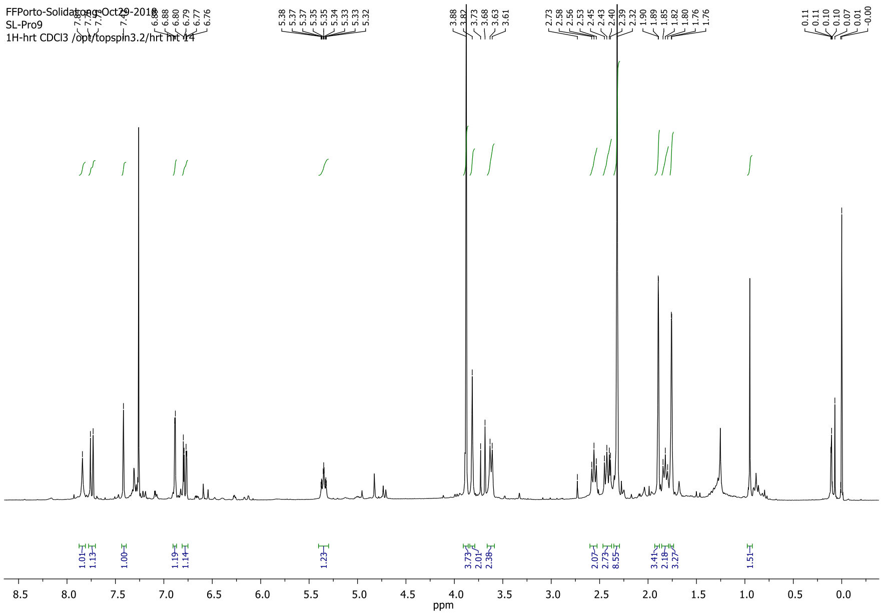

Figure S14: ^{1}H NMR spectrum of *3-{[(3-(Dimethylamino)propyl)(methyl)amino]methyl}-7-methoxy-1-(3-methylbut-2-en-1-yl)-9H-carbazol-2-ol (**2a**)* $CDCl_3$, 300, MHz).

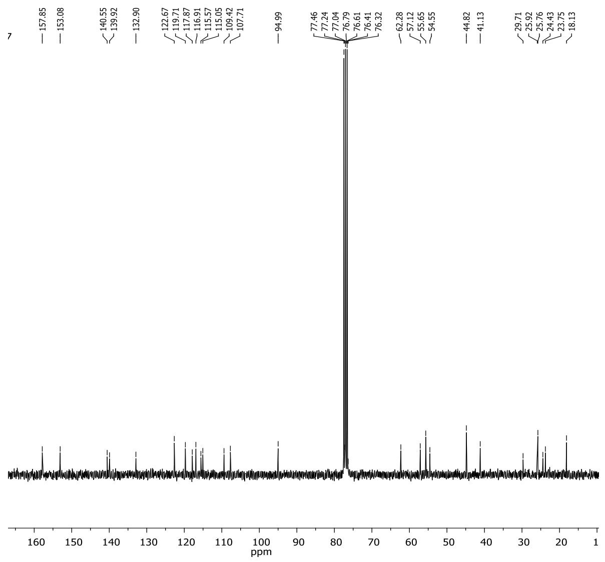

Figure S15: ^{13}C NMR spectrum of *3-{[(3-(Dimethylamino)propyl)(methyl)amino]methyl}-7-methoxy-1-(3-methylbut-2-en-1-yl)-9H-carbazol-2-ol (2**a**)* (CDCl$_3$, 75, MHz).

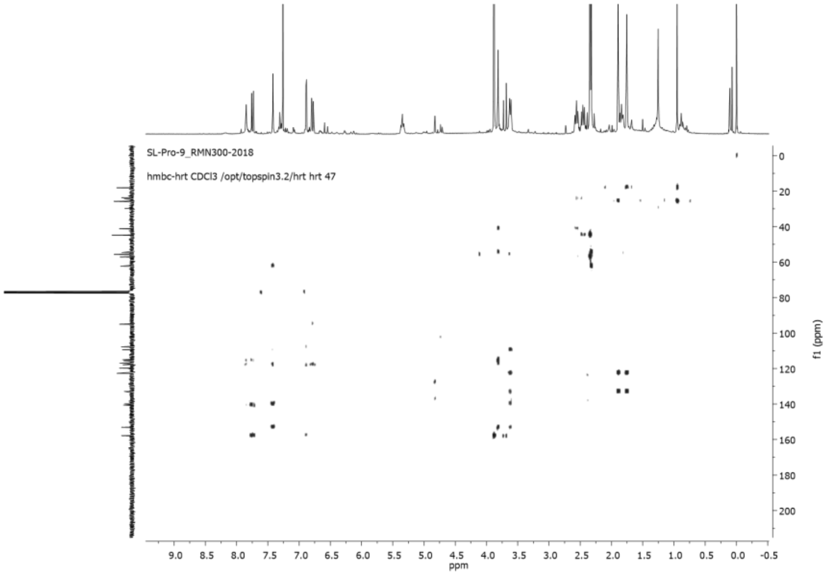

Figure S16: HRMS spectrum of *3-{[(3-(Dimethylamino)propyl)(methyl)amino]methyl}-7-methoxy-1-(3-methylbut-2-en-1-yl)-9H-carbazol-2-ol **(2a)*** (CDCl$_3$, 300, MHz).

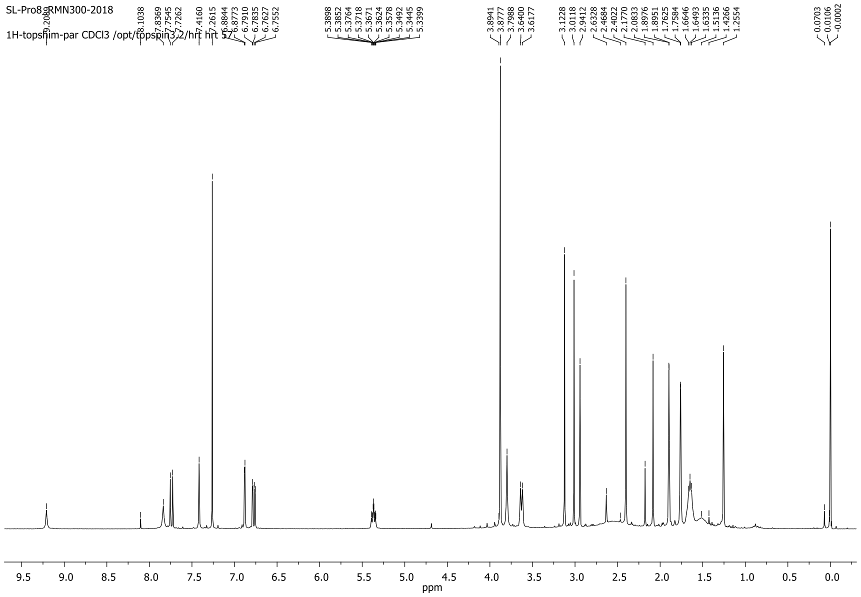

Figure S17: ^{1}H NMR spectrum of *7-Methoxy-1-(3-methylbut-2-en-1-yl)-3-(piperidin-1-ylmethyl)-9H-carbazol-2-ol (**2b**)*. (CDCl$_3$, 300, MHz).

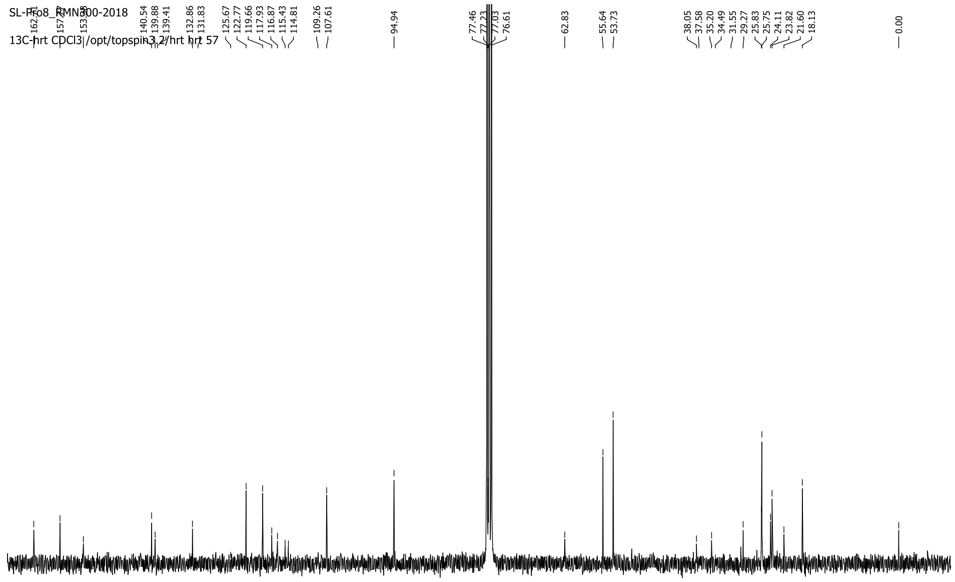

Figure S18: ^{13}C NMR spectrum of *7-Methoxy-1-(3-methylbut-2-en-1-yl)-3-(piperidin-1-ylmethyl)-9H-carbazol-2-ol (**2b**)*. (CDCl$_3$, 75, MHz).

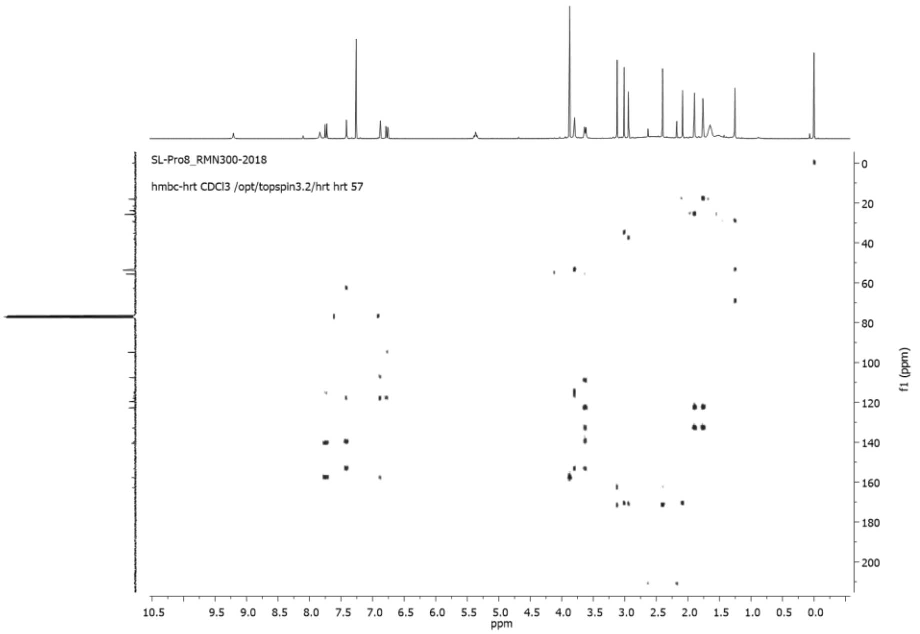

Figure S19: HMBC spectrum of *7-Methoxy-1-(3-methylbut-2-en-1-yl)-3-(piperidin-1-ylmethyl)-9H-carbazol-2-ol (**2b**)*. ($CDCl_3$, 300, MHz).

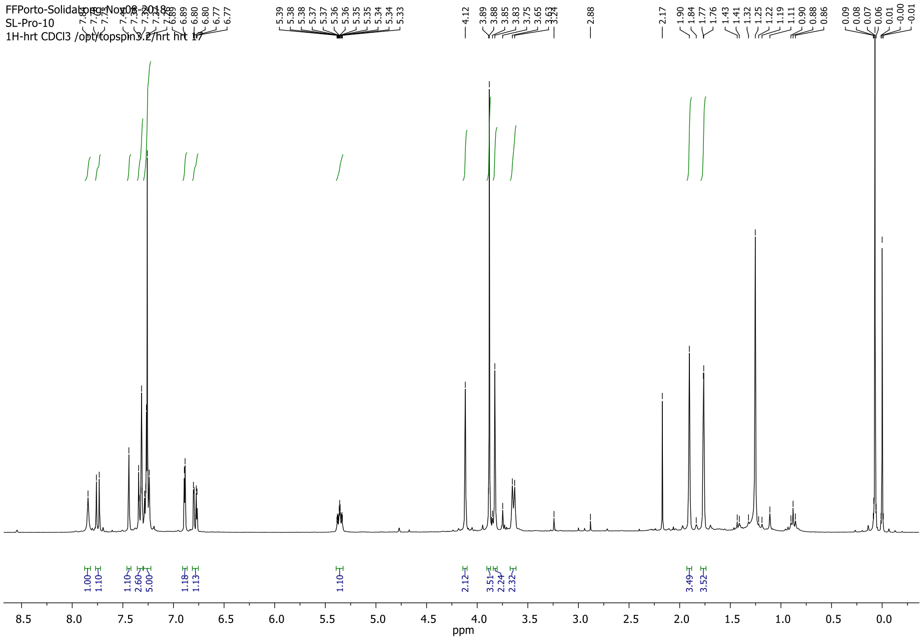

Figure S20: ^{1}H NMR spectrum of *3-{[(4-Chlorobenzyl)amino]methyl}-7-methoxy-1-(3-methylbut-2-en-1-yl)-9H-carbazol-2-ol (**2c**)*, ($CDCl_3$, 300, MHz).

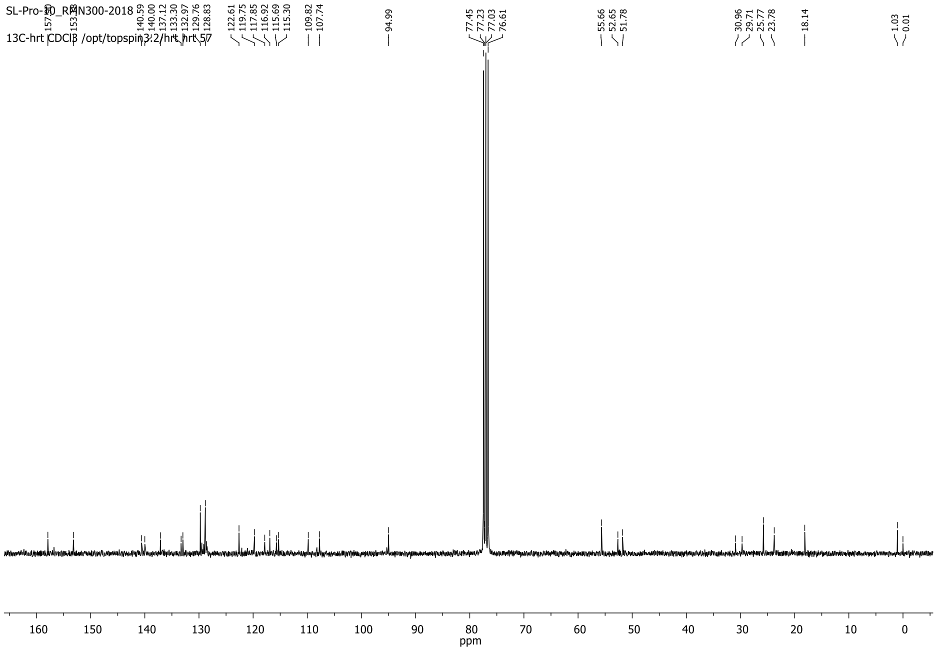

Figure S21: ^{13}C NMR spectrum of *3-{[(4-Chlorobenzyl)amino]methyl}-7-methoxy-1-(3-methylbut-2-en-1-yl)-9H-carbazol-2-ol (**2c**)* (CDCl$_3$, 75, MHz).

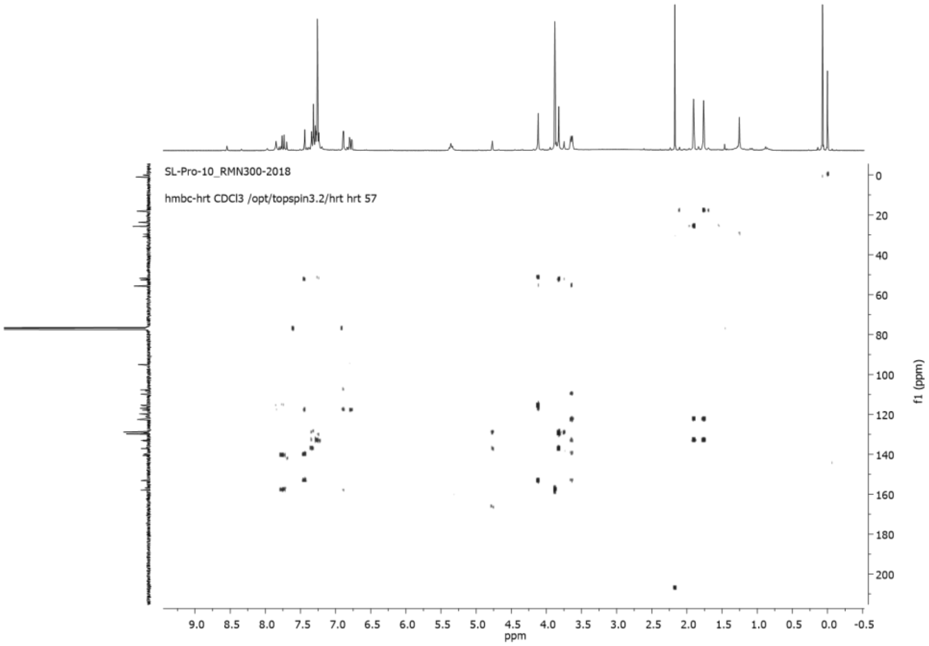

Figure S22: HMBC spectrum of *3-{[(4-Chlorobenzyl)amino]methyl}-7-methoxy-1-(3-methylbut-2-en-1-yl)-9H-carbazol-2-ol (**2c**)* (CDCl$_3$, 300, MHz).

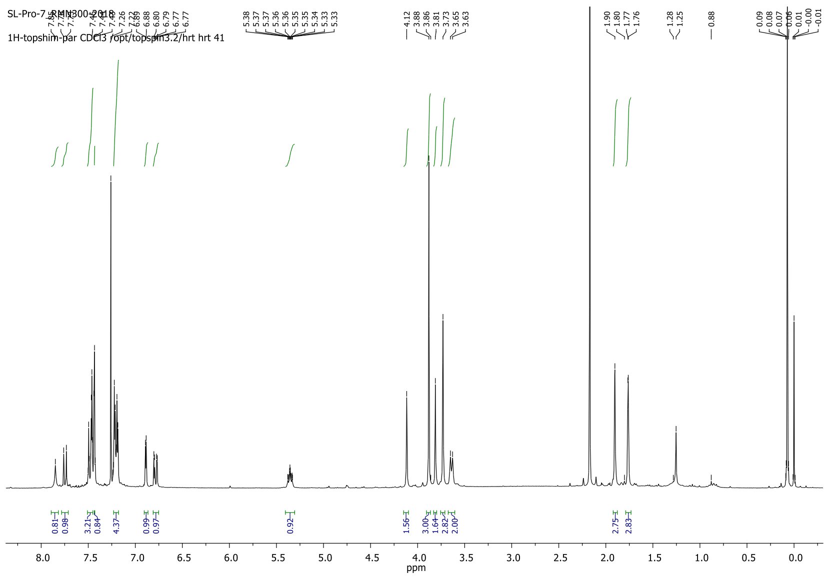

Figure S23: ^{1}H NMR spectrum of *3-{[(4-Fluorobenzyl)amino]methyl}-7-methoxy-1-(3-methylbut-2-en-1-yl)-9H-carbazol-2-ol (**2d**)* ($CDCl_3$, 300, MHz).

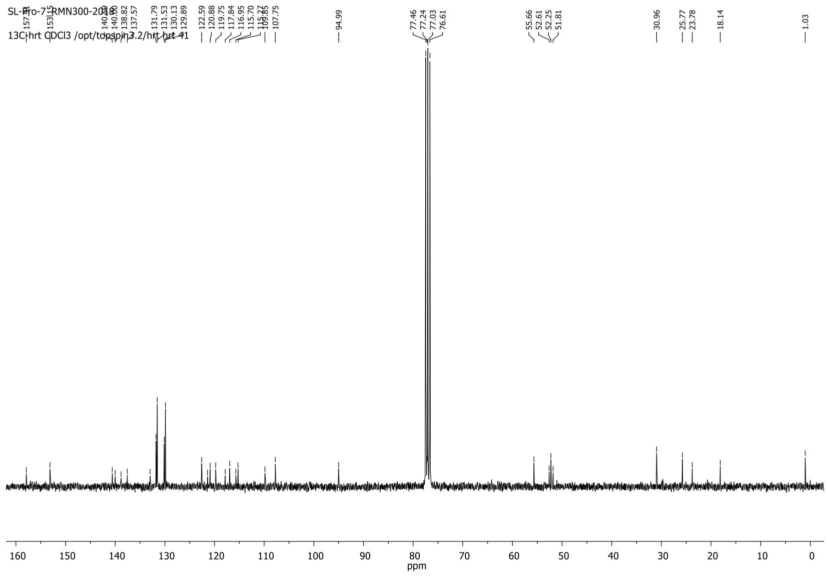

Figure S24: ^{13}C NMR spectrum of *3-{[(4-Fluorobenzyl)amino]methyl}-7-methoxy-1-(3-methylbut-2-en-1-yl)-9H-carbazol-2-ol (**2d**)* ($CDCl_3$, 75, MHz).

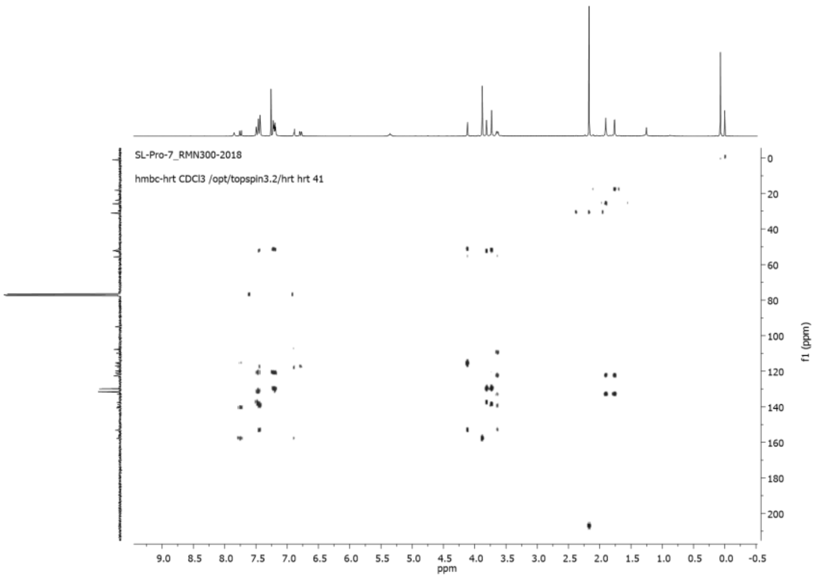

Figure S25: HMBC spectrum of *3-{[(4-Fluorobenzyl)amino]methyl}-7-methoxy-1-(3-methylbut-2-en-1-yl)-9H-carbazol-2-ol (**2d**)* ($CDCl_3$, 300, MHz).

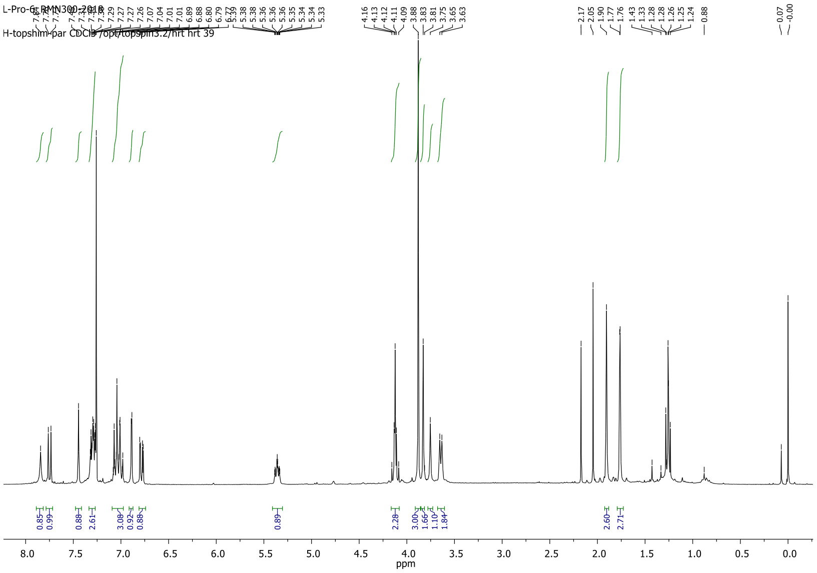

Figure S26: ^{1}H NMR spectrum *3-{[(4-Bromobenzyl)amino]methyl}-7-methoxy-1-(3-methylbut-2-en-1-yl)-9H-carbazol-2-ol (**2e**)* ($CDCl_3$, 300, MHz).

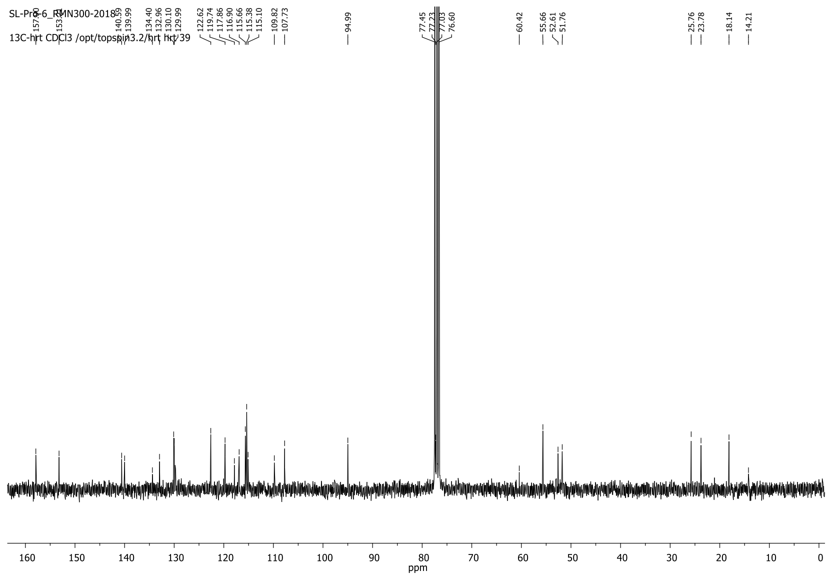

Figure S27: ^{13}C NMR spectrum of *3-{[(4-Bromobenzyl)amino]methyl}-7-methoxy-1-(3-methylbut-2-en-1-yl)-9H-carbazol-2-ol (**2e**)* ($CDCl_3$, 75, MHz).

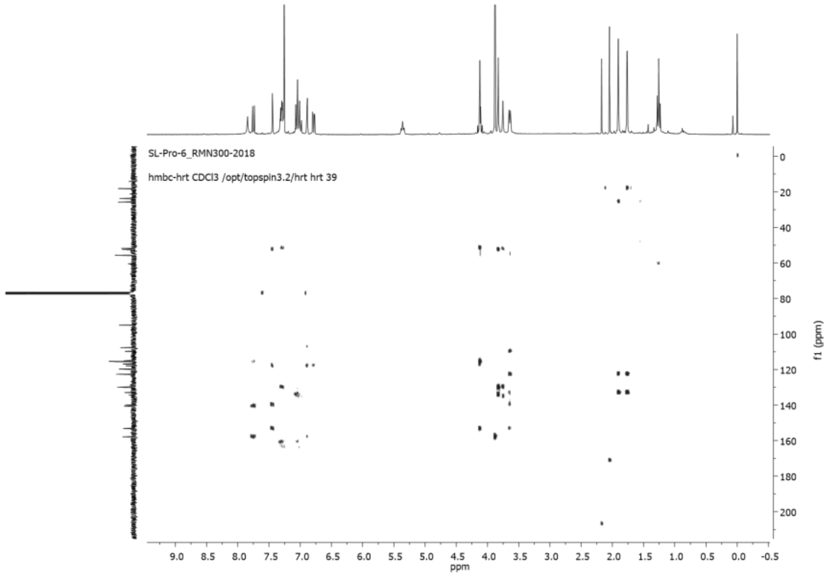

Figure S28: ^{13}C NMR spectrum of *3-{[(4-Bromobenzyl)amino]methyl}-7-methoxy-1-(3-methylbut-2-en-1-yl)-9H-carbazol-2-ol (**2e**)* ($CDCl_3$, 300, MHz).

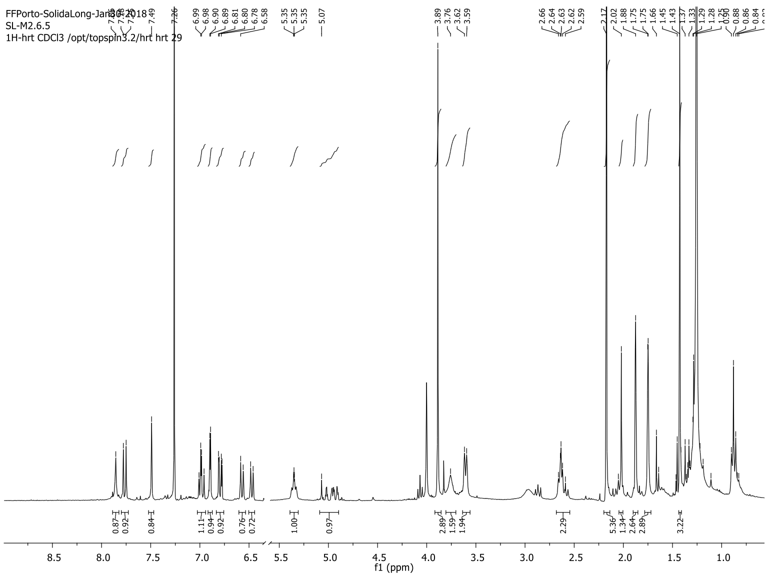

Figure S29: ^{1}H NMR spectrum of *3-[(5-Amino-3,4-dihydroisoquinolin-2(1H)-yl)methyl]-7-methoxy-1-(3-methylbut-2-en-1-yl)-9H-carbazol-2-ol (**2f**)*. (CDCl$_3$, 300, MHz).

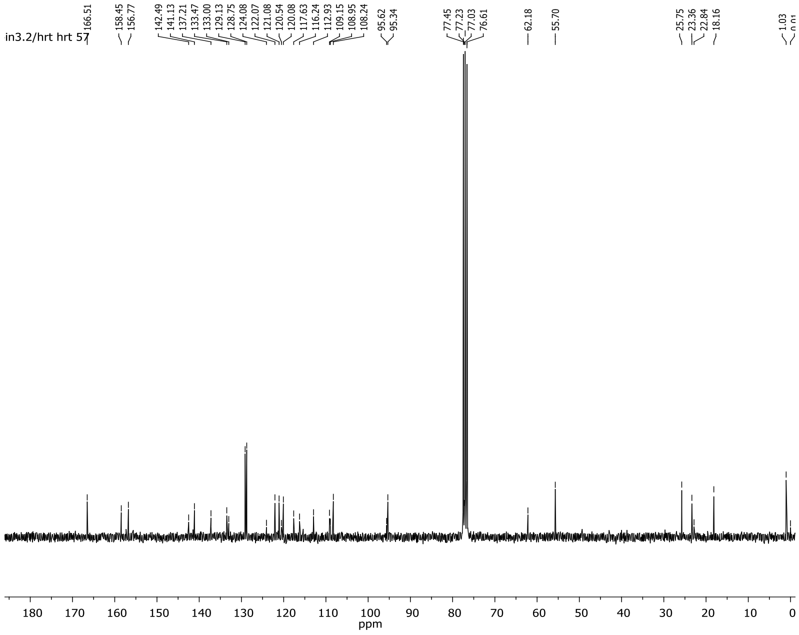

Figure S30: ^{13}C NMR spectrum of *3-[(5-Amino-3,4-dihydroisoquinolin-2(1H)-yl)methyl]-7-methoxy-1-(3-methylbut-2-en-1-yl)-9H-carbazol-2-ol (**2f**)*. (CDCl$_3$, 75, MHz).

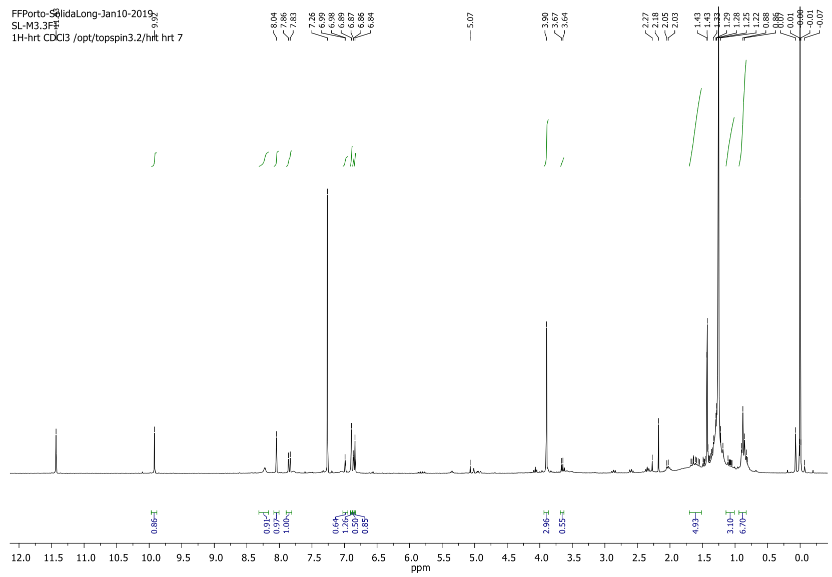

Figure S31: ^{1}H NMR spectrum of *3-{[(3-(Dimethylamino)propyl)(methyl)amino]methyl}-7-methoxy-9H-carbazol-2-ol (**3a**)* ($CDCl_3$, 300, MHz).

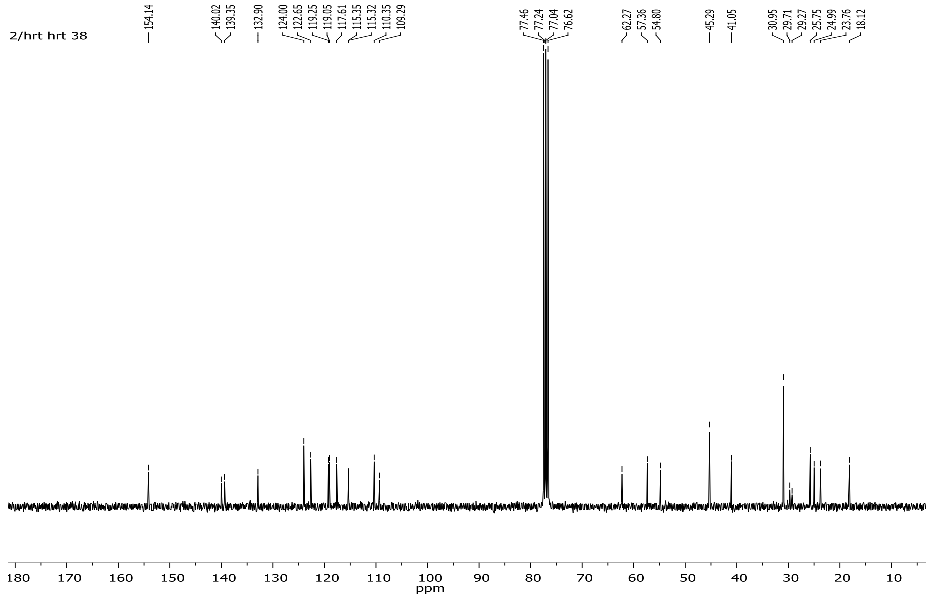

Figure S32: ^{13}C NMR spectrum of *3-{[(3-(Dimethylamino)propyl)(methyl)amino]methyl}-7-methoxy-9H-carbazol-2-ol (**3a**)* ($CDCl_3$, 75, MHz).

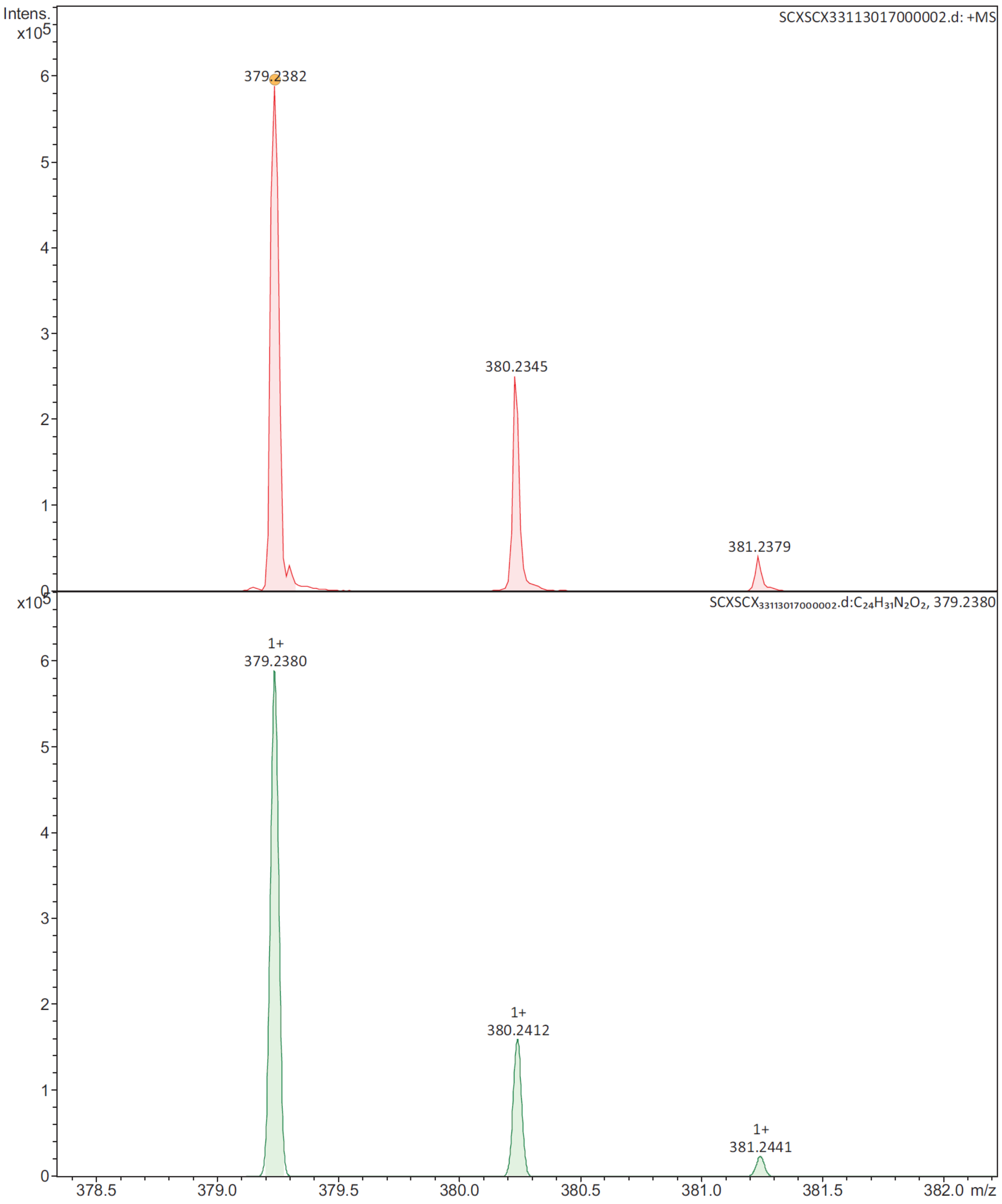

Figure S33: HRMS of compound *3-{[(3-(Dimethylamino)propyl)(methyl)amino]methyl}-1-(3-methylbut-2-en-1-yl)-9H-carbazol-2-ol* (***1a***), (20, 300 V).

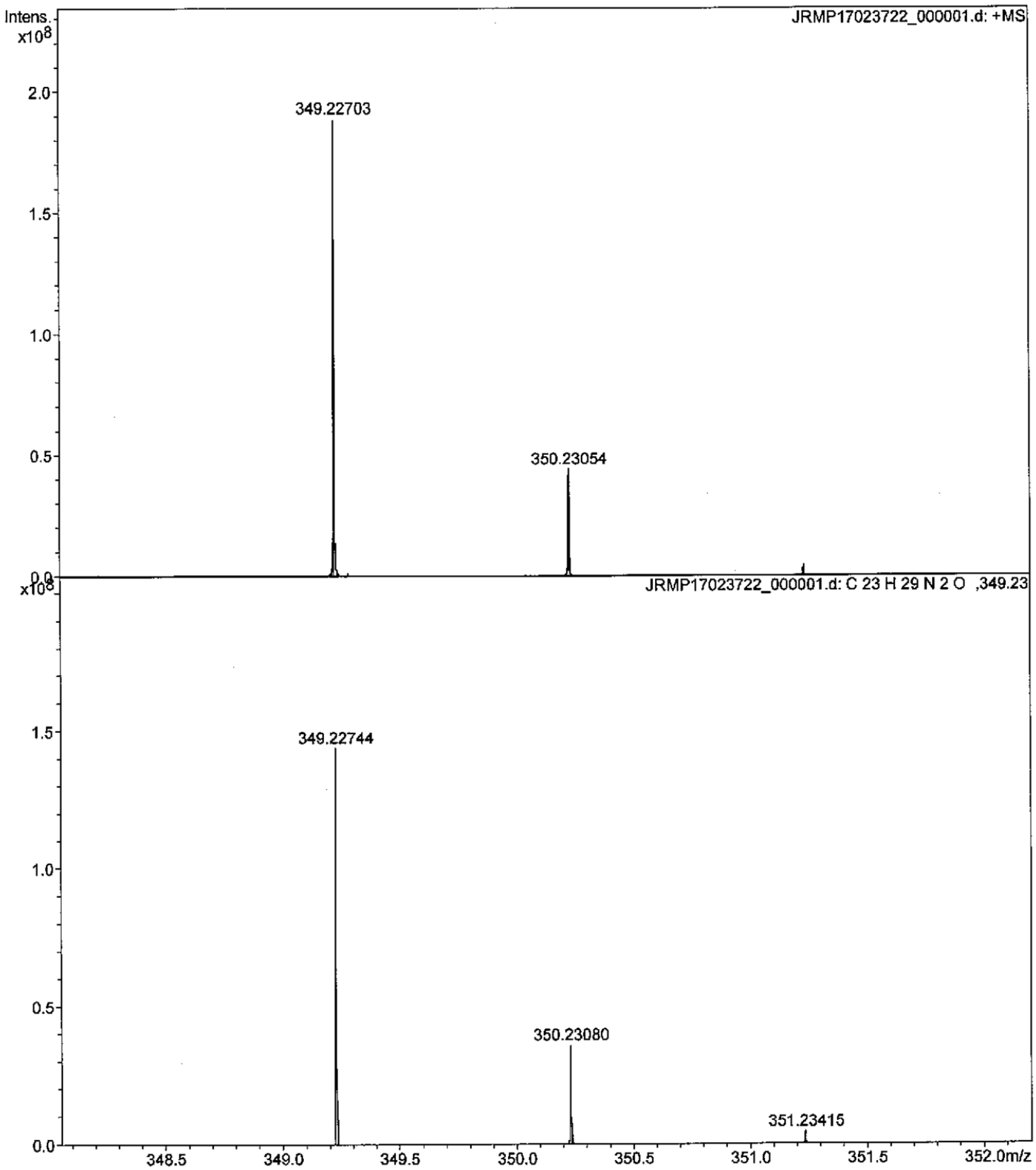

Figure S34: HRMS of compound *1-(3-Methylbut-2-en-1-yl)-3-(piperidin-1-ylmethyl)-9H-carbazol-2-ol* (***1b***), (20, 300 V).

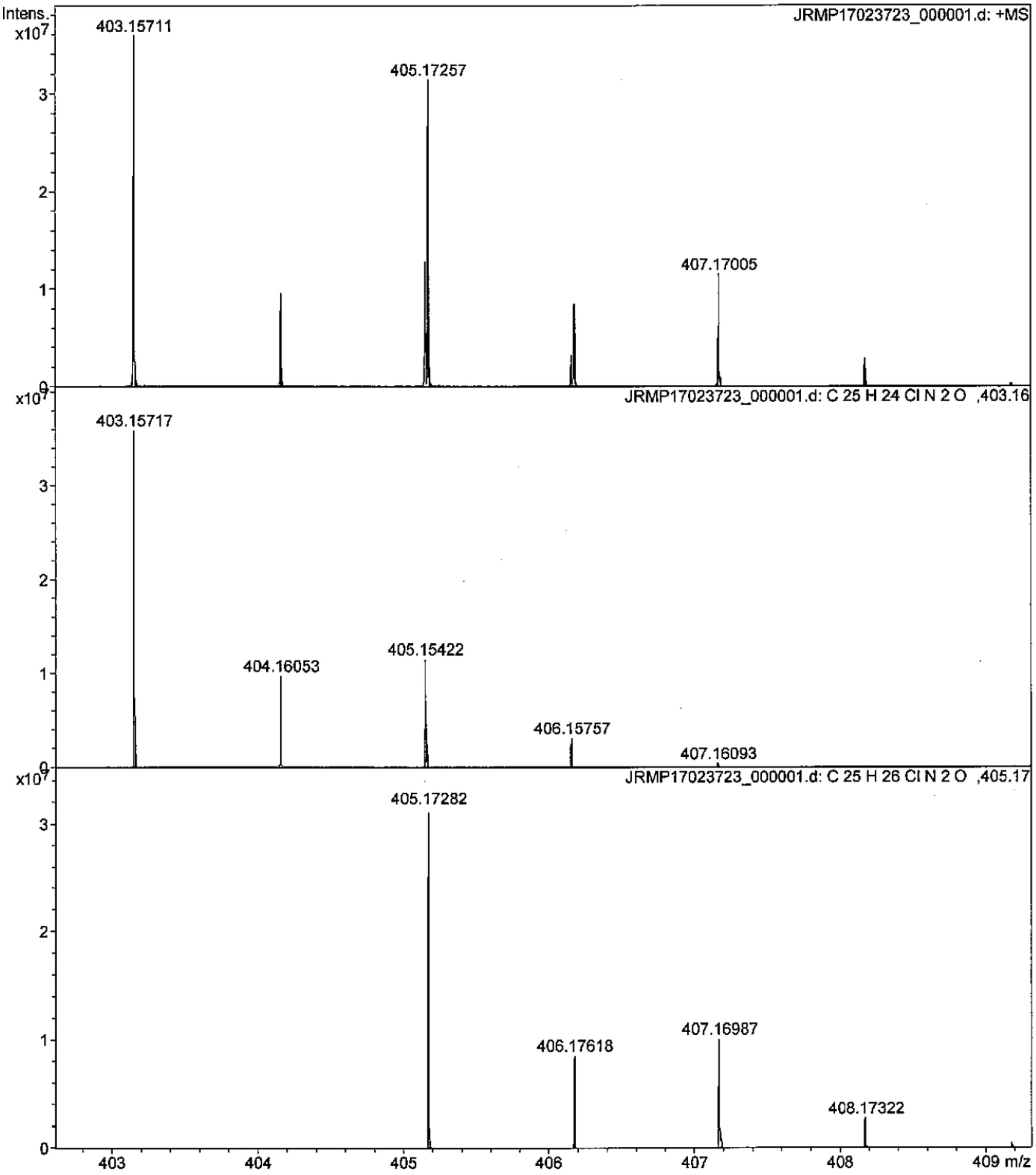

Figure S35: HRMS of compound of compound *3-{[(4-Chlorobenzyl)amino]methyl}-1-(3-methylbut-2-en-1-yl)-9H-carbazol-2-ol (**1c**),* (20, 300 V).

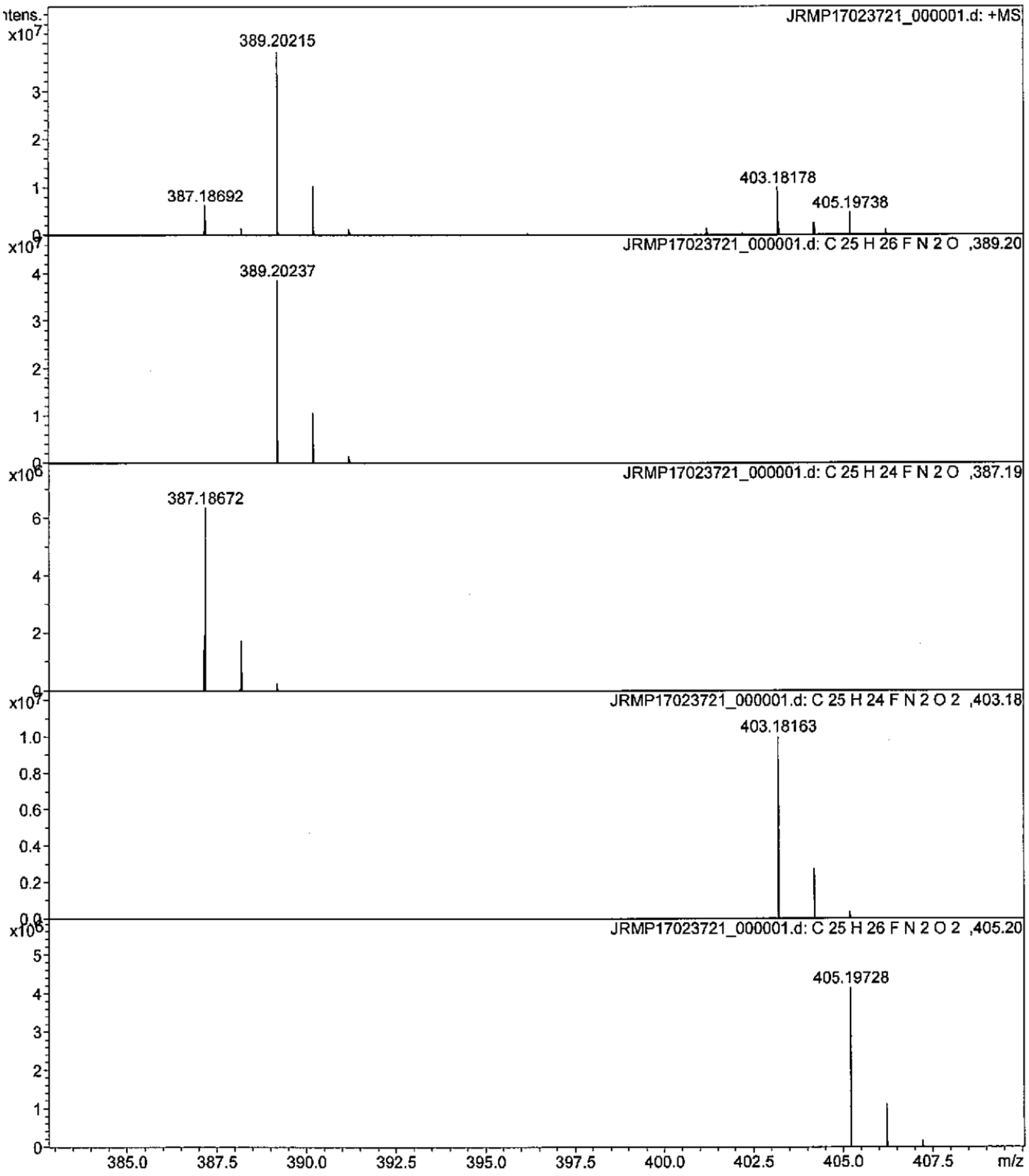

Figure S36: HRMS of compound *3-{[(4-Fluorobenzyl)amino]methyl}-1-(3-methylbut-2-en-1-yl)-9H-carbazol-2-ol* ***(1d)*** *(*20, 300 V).

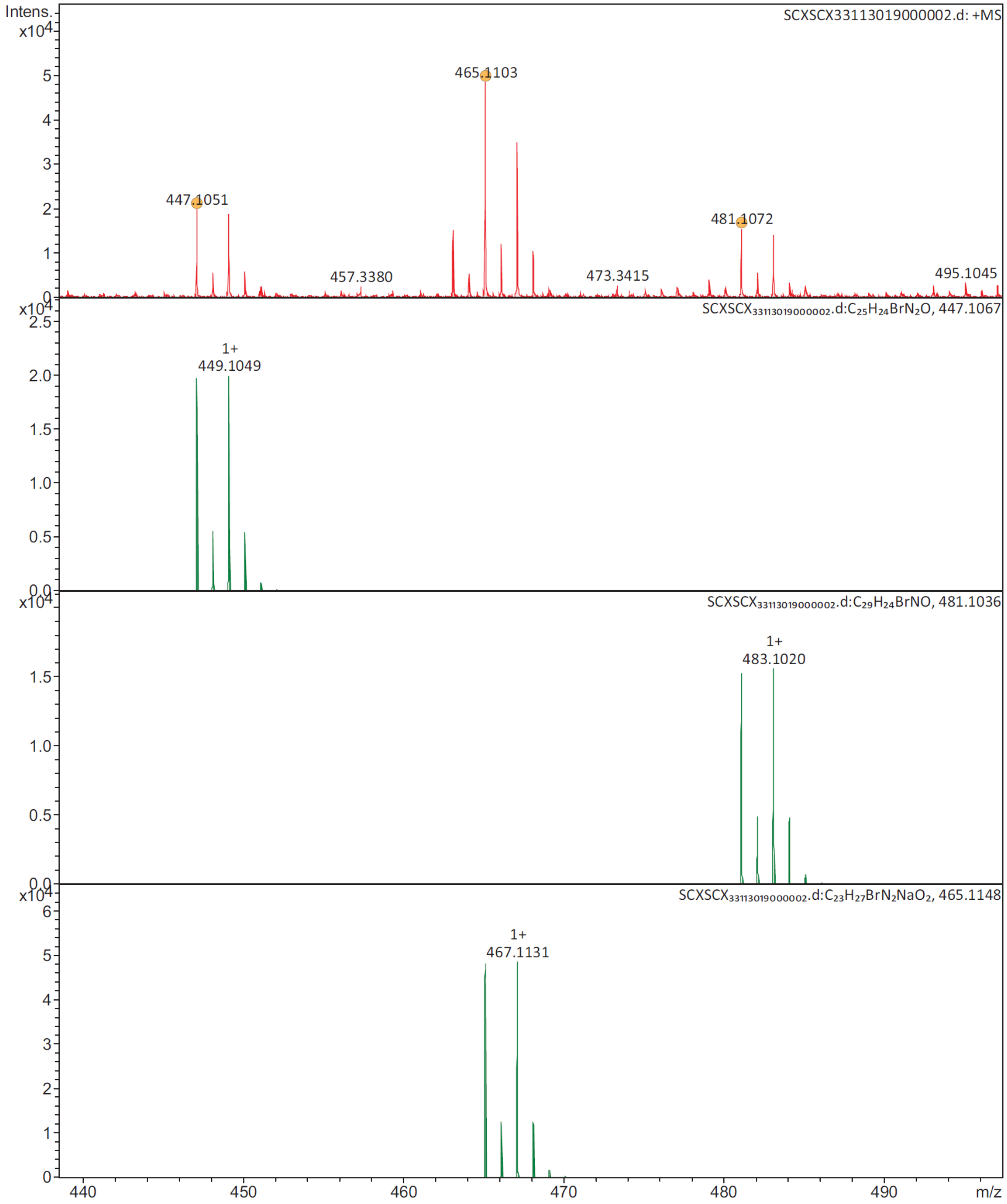

Figure S37: HRMS of compound *3-{[(4-Bromobenzyl)amino]methyl}-1-(3-methylbut-2-en-1-yl)-9H-carbazol-2-ol (**1e**)*. (20, 300 V).

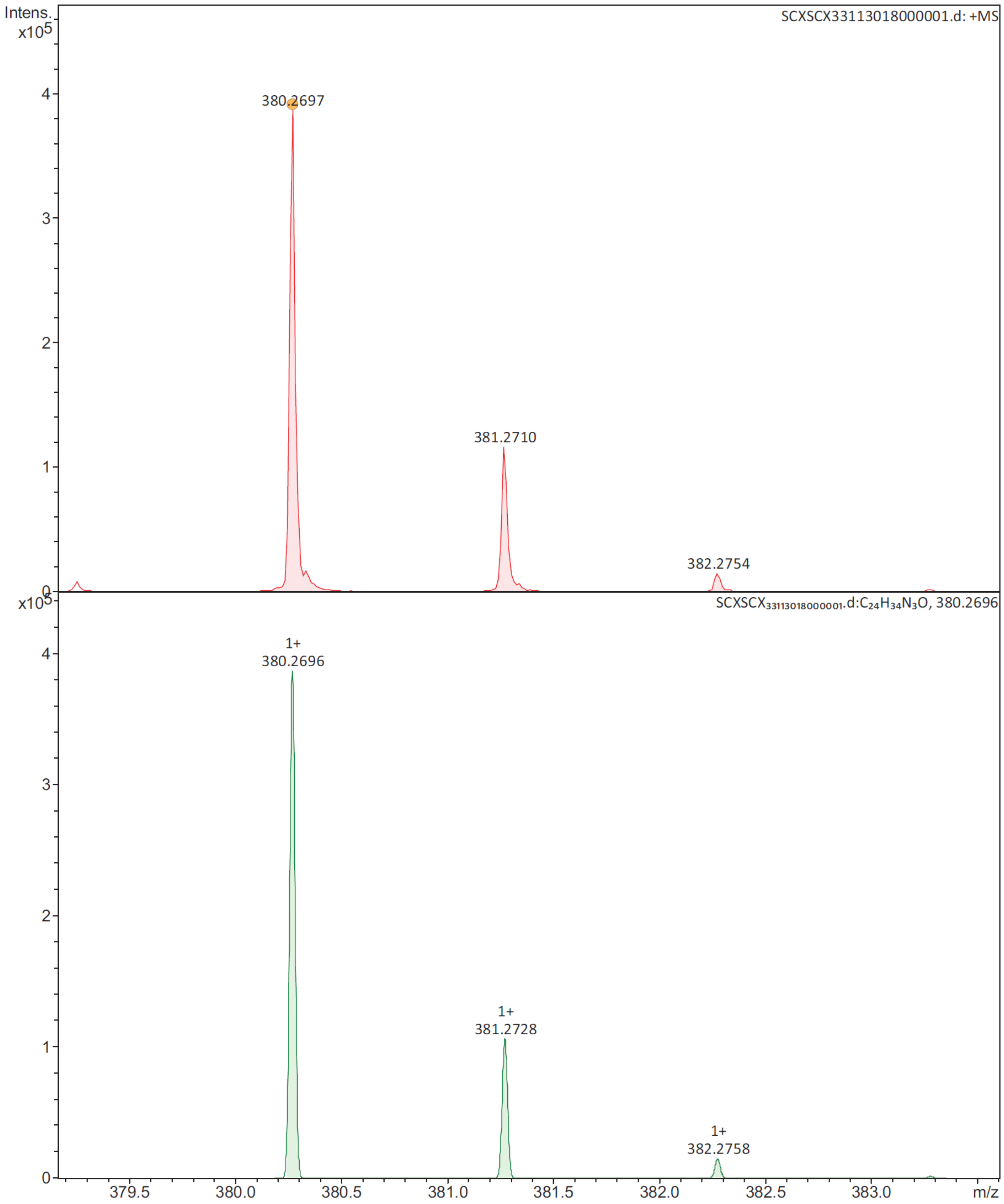

Figure S38: HRMS of compound *3-{[(3-(Dimethylamino)propyl)(methyl)amino]methyl}-7-methoxy-1-(3-methylbut-2-en-1-yl)-9H-carbazol-2-ol (**2a**)*, (20, 300 V).

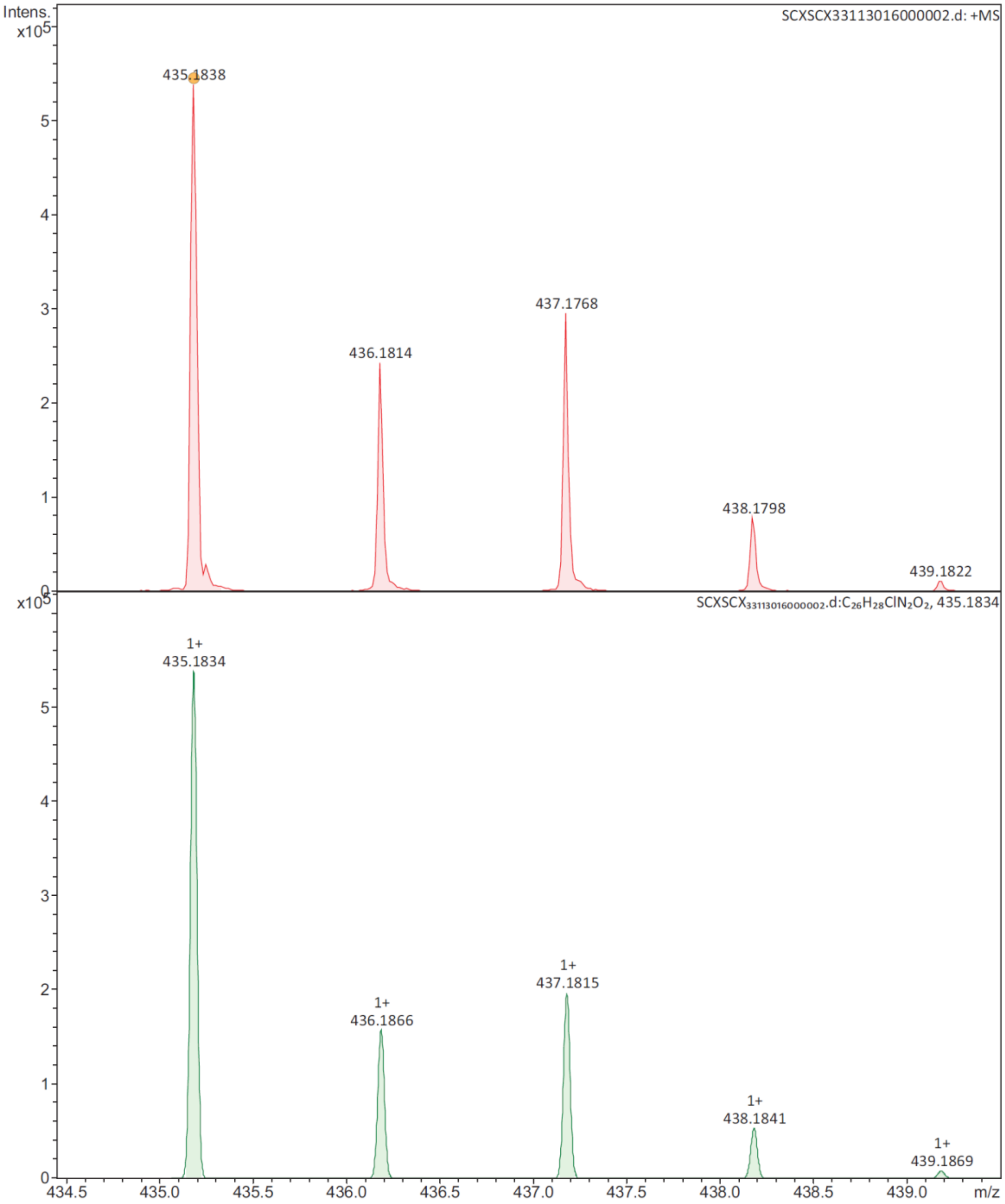

Figure S39: HRMS of compound *3-{[(4-Chlorobenzyl)amino]methyl}-7-methoxy-1-(3-methylbut-2-en-1-yl)-9H-carbazol-2-ol (**2c**)*, (20, 300 V).

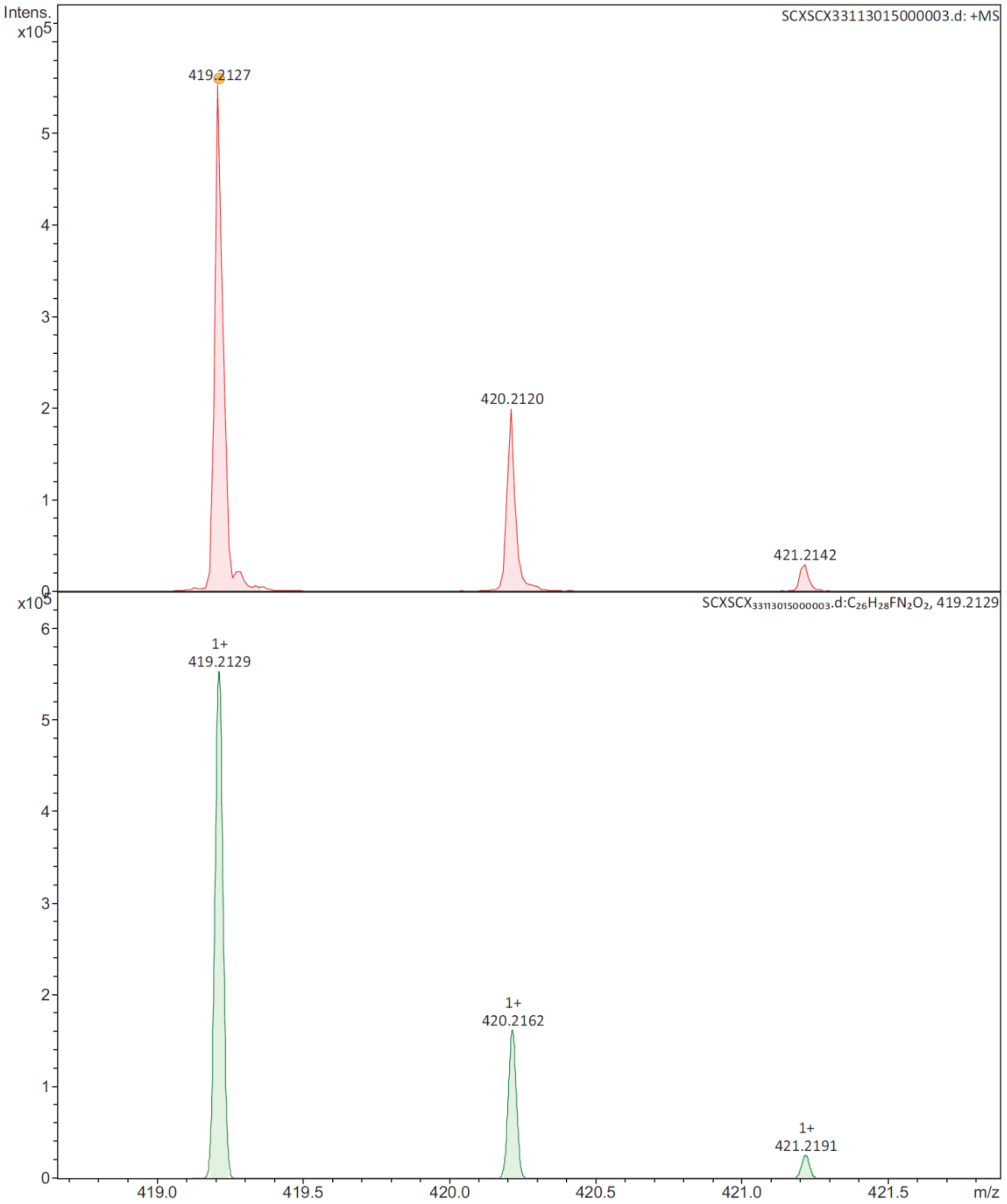

Figure S40: HRMS of compound *3-{[(4-Fluorobenzyl)amino]methyl}-7-methoxy-1-(3-methylbut-2-en-1-yl)-9H-carbazol-2-ol (**2d**)*, (20, 300 V).

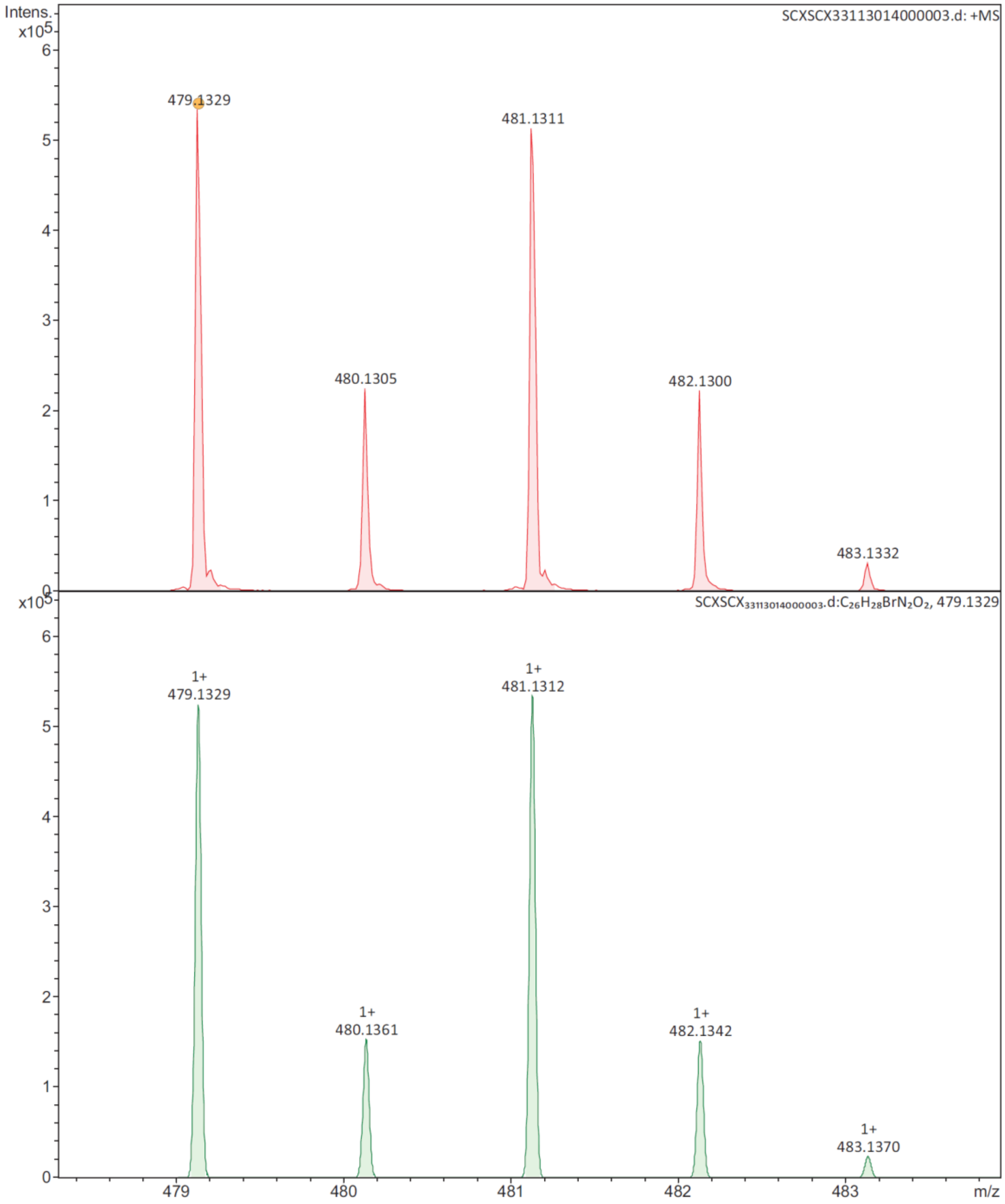

Figure S41: HRMS of compound *3-{[(4-Bromobenzyl)amino]methyl}-7-methoxy-1-(3-methylbut-2-en-1-yl)-9H-carbazol-2-ol (**2e**)*, (20, 300 V).

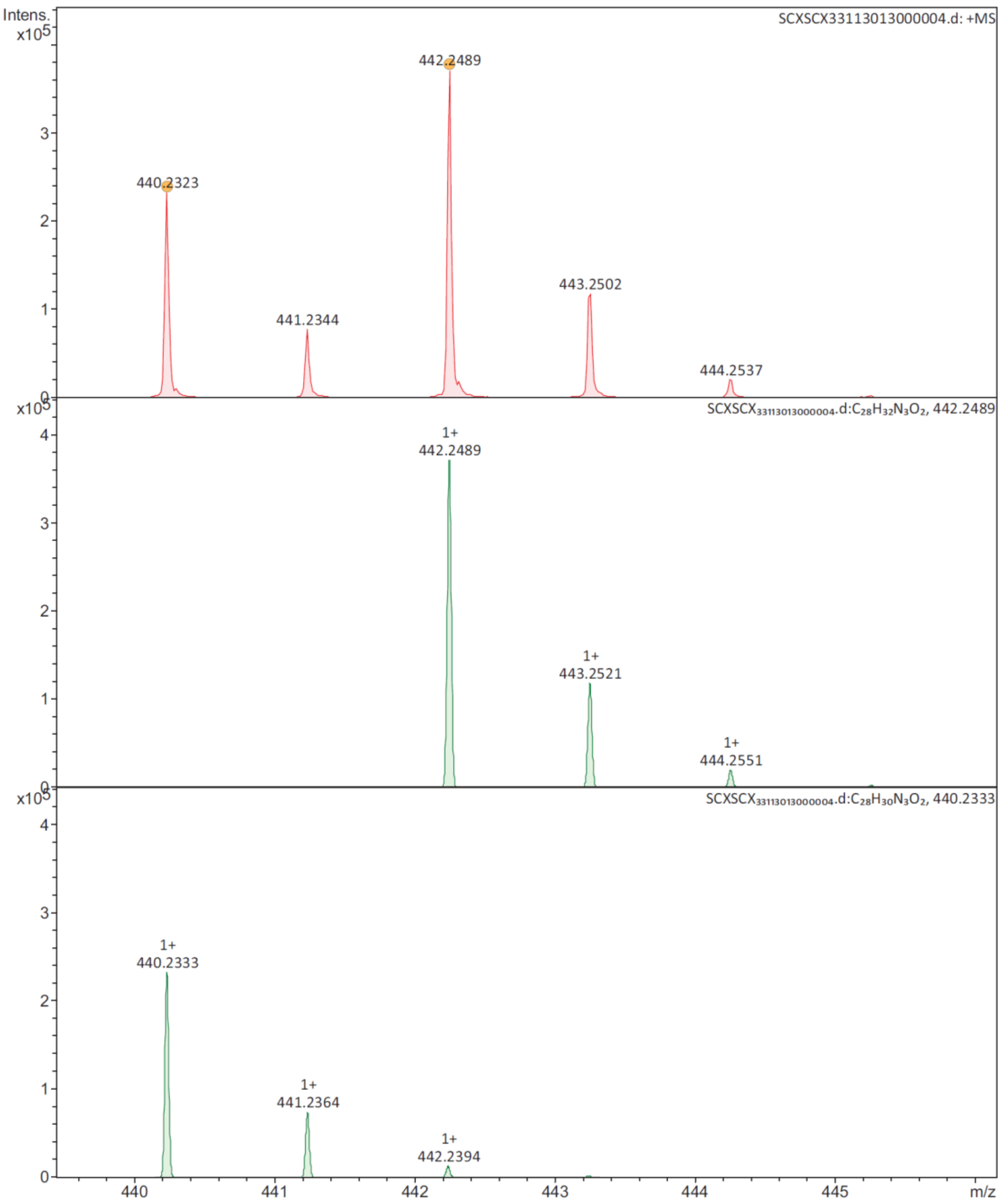

Figure S42: HRMS of compound *3-[(5-Amino-3,4-dihydroisoquinolin-2(1H)-yl)methyl]-7-methoxy-1-(3-methylbut-2-en-1-yl)-9H-carbazol-2-ol* (***2f***), (20, 300 V)

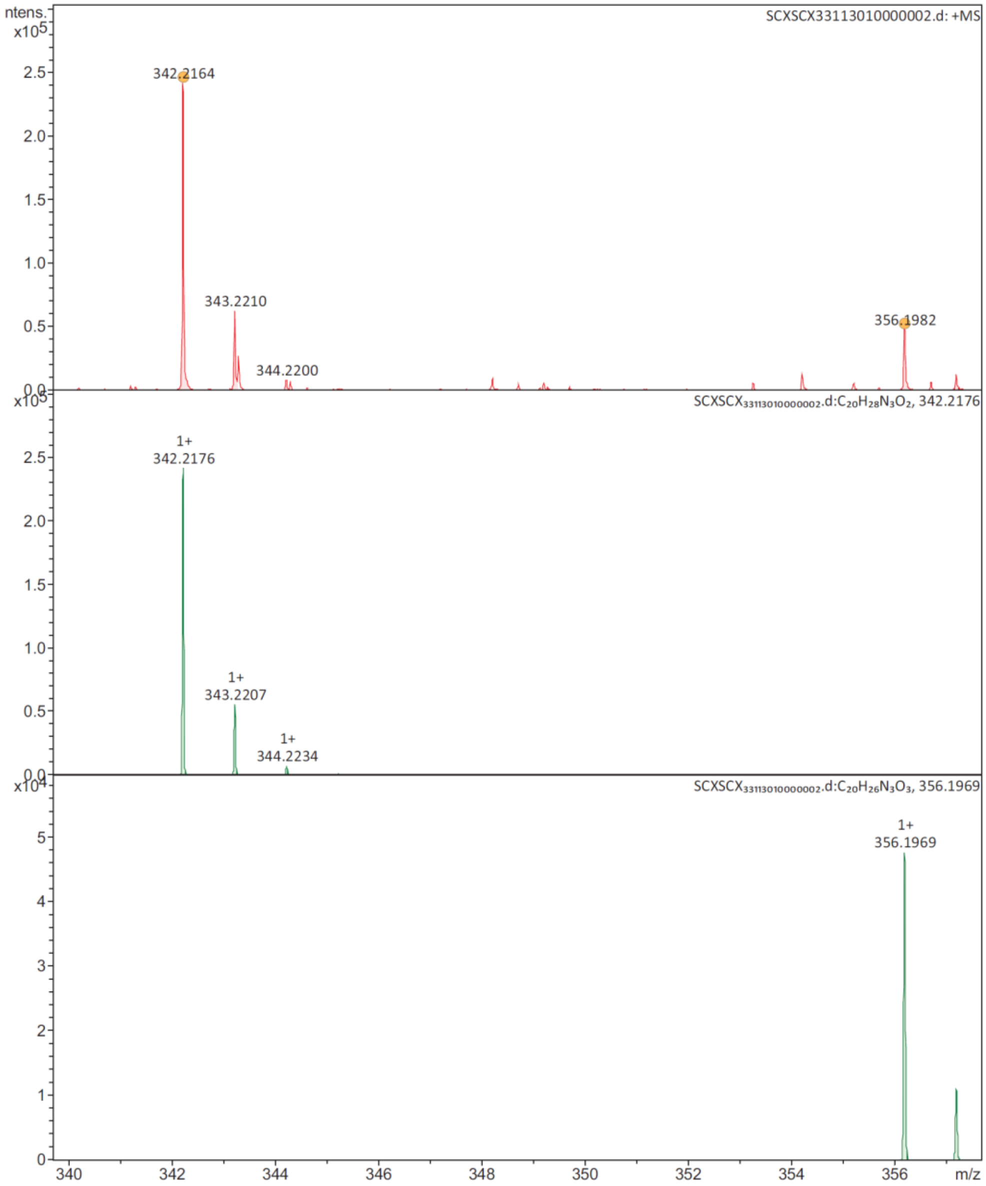

Figure S43: HRMS of compound *3-{[(3-(Dimethylamino)propyl)(methyl)amino]methyl}-7-methoxy-9H-carbazol-2-ol (**3a**),* (20, 300 V).
